# Critical temperature shift modeling of confined fluids using pore-size-dependent energy parameter of potential function

**DOI:** 10.1038/s41598-023-31998-7

**Published:** 2023-03-24

**Authors:** Mohammad Humand, Mohammad Reza Khorsand Movaghar

**Affiliations:** Department of Petroleum Engineering, Amirkabir University of Technology, No. 424, Hafez Ave., Tehran, Iran

**Keywords:** Thermodynamics, Chemical engineering, Statistical mechanics, Phase transitions and critical phenomena, Thermodynamics

## Abstract

The behavior and critical properties of fluids confined in nanoscale porous media differ from those of bulk fluids. This is well known as critical shift phenomenon or pore proximity effect among researchers. Fundamentals of critical shift modeling commenced with developing equations of state (EOS) based on the Lennard–Jones (L–J) potential function. Although these methods have provided somewhat passable predictions of pore critical properties, none represented a breakthrough in basic modeling. In this study, a cubic EOS is derived in the presence of adsorption for Kihara fluids, whose attractive term is a function of temperature. Accordingly, the critical temperature shift is modeled, and a new adjustment method is established in which, despite previous works, the bulk critical conditions of fluids are reliably met with a thermodynamic basis and not based on simplistic manipulations. Then, based on the fact that the macroscopic and microscopic theories of corresponding states are related, an innovative idea is developed in which the energy parameter of the potential function varies with regard to changes in pore size, and is not taken as a constant. Based on 94 available data points of critical shift reports, it is observed that despite L–J, the Kihara potential has sufficient flexibility to properly fit the variable energy parameters, and provide valid predictions of phase behavior and critical properties of fluids. Finally, the application of the proposed model is examined by predicting the vapor–liquid equilibrium properties of a ternary system that reduced the error of the L–J model by more than 6%.

## Introduction

In recent years, countless research works have been conducted in order to understand and evaluate the phase behavior of fluids confined in molecular scale pores, especially hydrocarbons, due to the growing demand for production from unconventional oil and gas reserves. Phase behavior and thermodynamics of confined fluids have a notable deviation from their corresponding bulk behavior. Experimental measurements^1–4^ have revealed that the thermodynamic and physicochemical properties of fluids, such as their critical properties, considerably shift when they are confined in a porous medium with pore radius of molecular scale. This phenomenon is originally named and known in the literature as Critical Shift or Pore Proximity Effect. Even its applicability has been investigated in several other research areas, such as nanofluidic chipset technologies^5^. Because of the occurrence of critical shift in confined media, applying the bulk properties of matters undoubtedly leads to poor results when calculating vapor–liquid equilibrium (VLE) properties of their confined state.

Various analytical and numerical methods have been examined to predict and model the above theory for pure normal fluids, e.g., developed microscopic van der Waals (vdW) equation of state (EOS) in a similar previous study^6^, molecular dynamics simulation (MD)^7^, density functional theory^8^, lattice Boltzmann method (LBM)^9^, scaled particle theory (SPT or RFL)^10^, and different classes of Monte Carlo simulations^11–19^.

Numerical approaches are frequently inconvenient to be applied to a different scope since they are accompanied by numerous limitations and great complexity. Having a theoretical and thermodynamic basis, the mentioned modified vdW EOS has been widely used because of its compatibility with experimental values on the one hand, and its ease of use on the other. Such a convenience would be much preferable to implementing time-consuming and inconvenient huge numerical simulation for every research case. The EOS method has been developed using the Lennard–Jones (L–J) potential function with two adjustable parameters (${\sigma }_{LJ}$, $\varepsilon /k$) whose values can be determined via least-square fitting of thermodynamic properties^20^. A three-parameter potential function (like Kihara), nevertheless, is supposed to perform much better due to possessing the ability to provide more flexible modeling and accurate predictions^21^.

In addition, fluid adsorptions and their huge impacts on phase behavior cannot be ignored^22,23^. A multi-layer film of fluid is always absorbed by pore walls and its contribution to fluid characteristics is great enough to be considered, because, in nanoscale (confined) porous media, the wall-molecule interactions are not insignificant anymore as they are in bulk state. In this regard, analytical^22–24^ and numerical^25–28^ research works have been conducted to include the adsorption phenomenon. Although experimental measurements are always highly reliable and give direct understanding of fluid properties, they are greatly time-consuming and require highly expensive setups to study confined porous media. Moreover, it is quite difficult to establish an apparatus that could last under extreme conditions of high pressure and temperature^23^.

Over the last few years, various measures have been taken to enhance the confinement modeling of fluids; yet, no fundamental and analytical development was presented except for the previous original model of L–J^6^. Other fellow researchers have merely manipulated numbers and coefficients through curve fittings^29–32^. In several works, furthermore, the mentioned L-J approach has been repeated with corrections of the theory’s coefficients^33–35^. These numerical enhancements strongly depend on the available data sets of one specific compound, and are probably inappropriate for comprehensive applications. Such uncertainty even led to developing exclusively curve-fitted correlations for substances like CH_4_, C_2_H_6_, and CO_2_^4,31^.

Despite Lennard–Jones (previous works), the Kihara potential function has three adjustable parameters and incorporates the hard-impenetrable core of a molecule into the modeling via its third potential parameter, ${a}_{k}$ ^36^. However, there is no limitation for two approaching molecules in the L-J model and they can completely penetrate. Kihara successfully fits thermodynamic data, specifically the second virial coefficient for different types of fluids. The L-J potential, in contrast, performs weaker in lower temperatures, whilst the flexibility of the Kihara potential makes it much easier to have a perfect fitting^20^.

**Figure 1:**
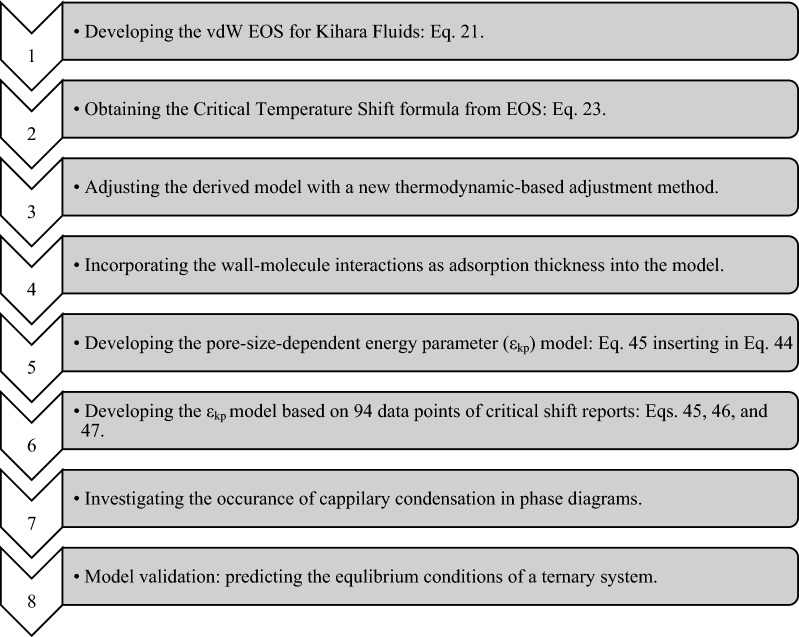
The general sketch of this study.

In the current study, first the theory is presented where the vdW EOS is derived for Kihara fluids as a generalized format of the Lennard–Jones. Despite previous works, the energy integral is solved exactly and without any approximations, leading to an exclusive solution for each fluid at any specific temperature. Second, an innovative idea is developed for the very first time in which the energy parameter of the potential is taken as a function of pore radius and is not constant. Third, this notion is supported by a collection of 94 critical shift data points available in the literature–80% for model development (training) and 20% for model verification (testing)–so as to make the phase behavior calculations much more reliable. Forth, the occurrence of capillary condensation is investigated for the van der Waals and Peng-Robinson equations of state when coupled with the Lennard–Jones and Kihara potential functions. Finally, the application of our study is investigated by phase behavior calculations in the last section where the performance of the proposed model is examined by calculating the vapor–liquid equilibrium (VLE) properties of a ternary mixture, and its results are compared to those of the L–J model and the experimental values. Figure 1 presents the general sketch of the studied problem in this work.

## Theory

### How to better model the critical temperature of confined fluids?

**Figure 2:**
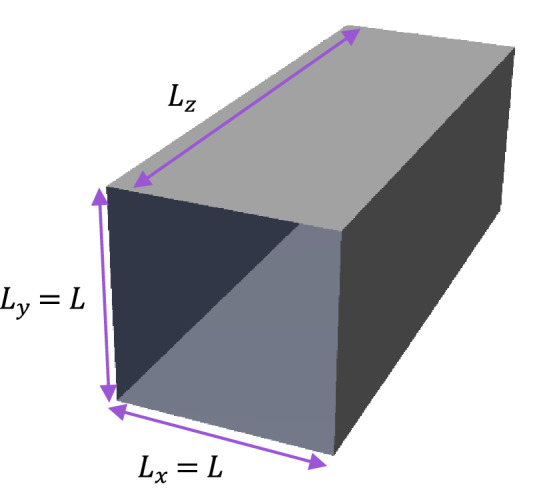
Schematic of a nano-scale pore in the Cartesian coordinates.

At the first step, the conventional vdW EOS must be modified in order to describe the confinement phenomenon. Although a simple cubic EOS, the vdW model is properly able to predict the vapor–liquid equilibrium (VLE) conditions and phase behavior properties of either pure or mixing fluids. As shown in Fig. 2, we consider a Cartesian pore model (${L}_{x}={L}_{y}$ and ${L}_{z}=\infty$) consisting of confined fluids for which the pressure $\overrightarrow{P}$ is a diagonal tensor with components ${p}_{ii},\left(i=x,y,z\right)$. The internal energy is given by^37^,

1 $$dE=TdS-{\sum }_{i}{p}_{ii}d{\epsilon }_{ii}V$$

where the term $-{\sum }_{i}{p}_{ii}d{\epsilon }_{ii}V$ shows how much work the internal tension has done under a specific deformation $d{\epsilon }_{ii}$ of volume $V$. From the Helmholtz free energy $F=E-TS$ we have^37^

2 $$dF=-SdT-{\sum }_{i}{p}_{ii}d{\epsilon }_{ii}V$$

Therefore the Helmholtz free energy of a system including N particles interacting by a pair potential $U\left({r}_{12}\right)$ can be derived from the perturbation theory as^6^

3 $$F=f\left(T\right)-\frac{kT{N}^{2}}{2{V}^{2}} \iint \left({e}^{-\beta U({r}_{12})}-1\right) d{V}_{1}d{V}_{2}$$

where $f\left(T\right)$ is the free energy of ideal gas; $k$ is the Boltzmann constant; and $\beta$ is $1/kT$. The term ${e}^{-\beta U({r}_{12})}-1$ is often called the Mayer function. Here, we utilize the Kihara potential with three adjustable parameters for the interaction between particles. But why Kihara? Its formulation (Eq. 4) is the generalized format of the Lennard–Jones; hence, L-J would be a special case of Kihara and its corresponding properties will be readily in hand when the Kihara hard core is ignored.

Despite the Lennard–Jones theory in which two molecules can fully interpenetrate provided that they have enough energy^20^, in the Kihara model, the repulsion interaction in the rigid spherical core is split into two parts; a rigid core and an outer soft spherical repulsion region^38^. We suppose soft-penetrable electron clouds surround a hard-impenetrable core. Given that, the model’s mathematical expression would be^20^,

4 $$U\left({r}_{12}\right)=\left\{\begin{array}{c}\infty \\ 4\varepsilon \left[{\left(\frac{{\sigma }_{k}-2{a}_{k}}{r-2{a}_{k}}\right)}^{12}-{\left(\frac{{\sigma }_{k}-2{a}_{k}}{r-2{a}_{k}}\right)}^{6}\right]\end{array}\right. \genfrac{}{}{0pt}{}{\begin{array}{c}r<2{a}_{k}\\ \end{array}}{r\ge 2{a}_{k}}$$

where ${a}_{k}$ is the radius of the spherical core; $\varepsilon$ is the depth of the energy well (herein we employ ${{\varvec{\varepsilon}}}_{{\varvec{k}}}=\varepsilon /k$ instead of $\varepsilon$); and ${\sigma }_{k}$ is the collision diameter, i.e., the distance $r$ between molecular centers when $U\left({r}_{12}\right)=0$. The intermolecular distance can go from ${r}_{12}=2{a}_{k}$ (where the repulsion force is infinity) to ${r}_{12}=\infty$.

Given Eq. (3), the standard vdW equation can be derived by integrating over an infinite volume. For a finite volume, the same procedure could be applied by splitting the integral into two regions, $2{a}_{k}<{r}_{12}<{\sigma }_{k}$ and ${r}_{12}>{\sigma }_{k}$:

5 $$F=f\left(T\right)-\frac{kT{N}^{2}}{2{V}^{2}} \left[\underset{2a<{r}_{12}<\sigma }{\overset{ }{\iint }}\left({e}^{-\beta U({r}_{12})}-1\right) d{V}_{1}d{V}_{2}+ \underset{{r}_{12}>\sigma }{\overset{ }{\iint }}\left({e}^{-\beta U({r}_{12})}-1\right) d{V}_{1}d{V}_{2}\right]$$

**Figure 3:**
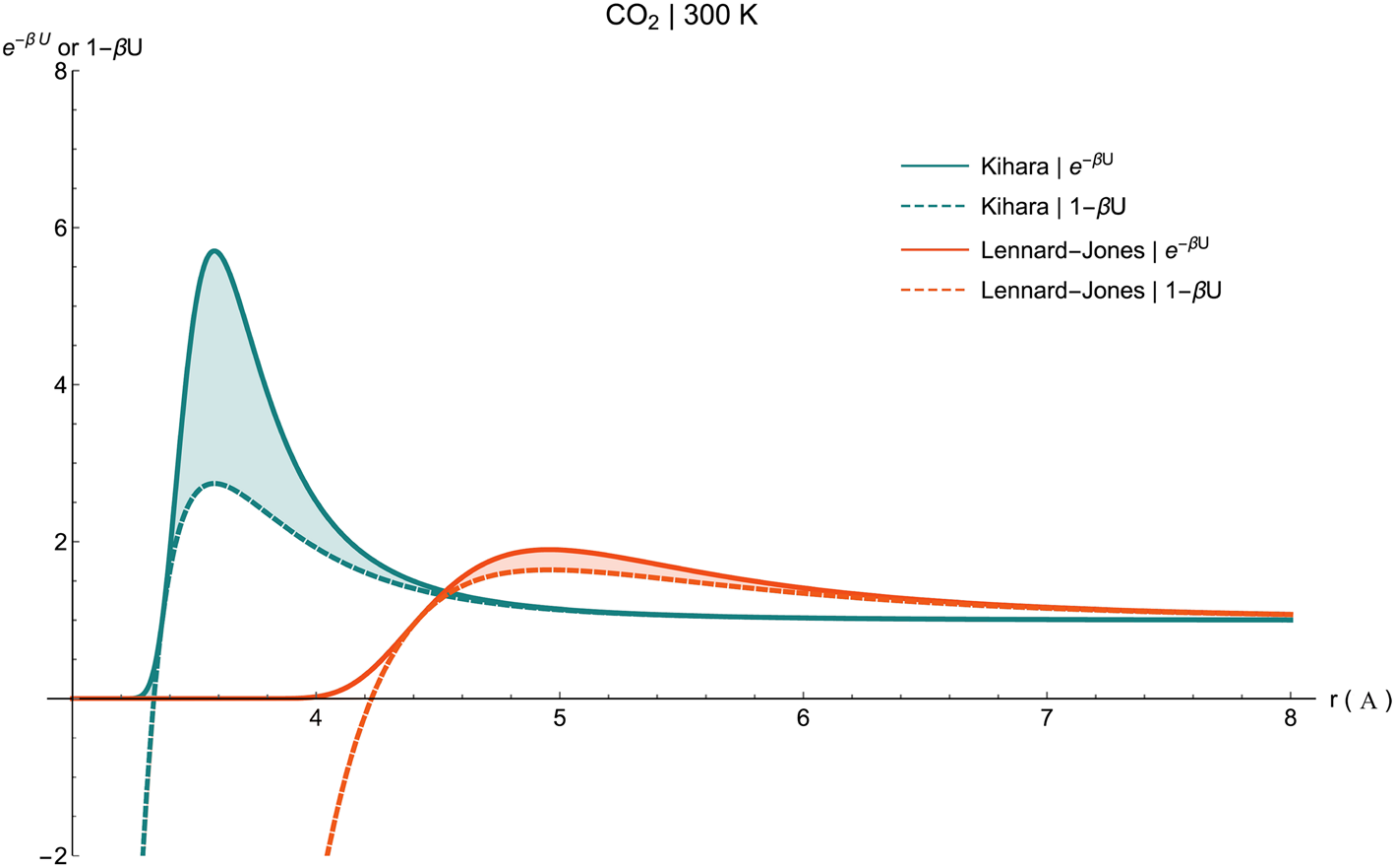
Comparison of the approximation terms between the Kihara and L-J models.

When the intermolecular distance is less than ${\sigma }_{k}$, the value of $U\left({r}_{12}\right)$ increases drastically to infinity; hence, we can utilize the assumption ${e}^{-\beta U\left({r}_{12}\right)}\cong 0$. Then the energy is given by,

6 $$F=f\left(T\right)+\frac{kT{N}^{2}}{V}b-\frac{kT{N}^{2}}{2{V}^{2}}\underset{{r}_{12}>\sigma }{\overset{ }{\iint }}\left(1-{e}^{-\beta U({r}_{12})}\right) d{V}_{1}d{V}_{2}$$

where $b=\frac{2\pi }{3}\left({\sigma }_{k}^{3}-{\left(2{a}_{k}\right)}^{3}\right)$ and $V={L}_{x}{L}_{y}{L}_{z}$ in which ${L}_{x}={L}_{y}=L$ and ${L}_{z}={L}_{z}$ for a pore model in the Cartesian coordinates. In spite of what has been done for the L–J^6^, here the latter integral will be solved exactly, without any simplifying assumption (henceforth referred to as “**exact solution**” for simplification). The reason is that the approximation term in the region ${r}_{12}>{\sigma }_{k}$, i.e., ${e}^{-\beta U({r}_{12})}\cong 1-\beta U({r}_{12})$, is in fact a good and close approximation for the L–J model, while such a substitution could not necessarily be made for the Kihara. Considering CO_2_ at 300 K an example, this assertion is well supported for both potentials in Fig. 3 where the light-colored region of L–J is insubstantial compared to that of Kihara. This difference is even more intense at lower temperatures, leaving no other alternatives except for the exact solution.

**Figure 4:**
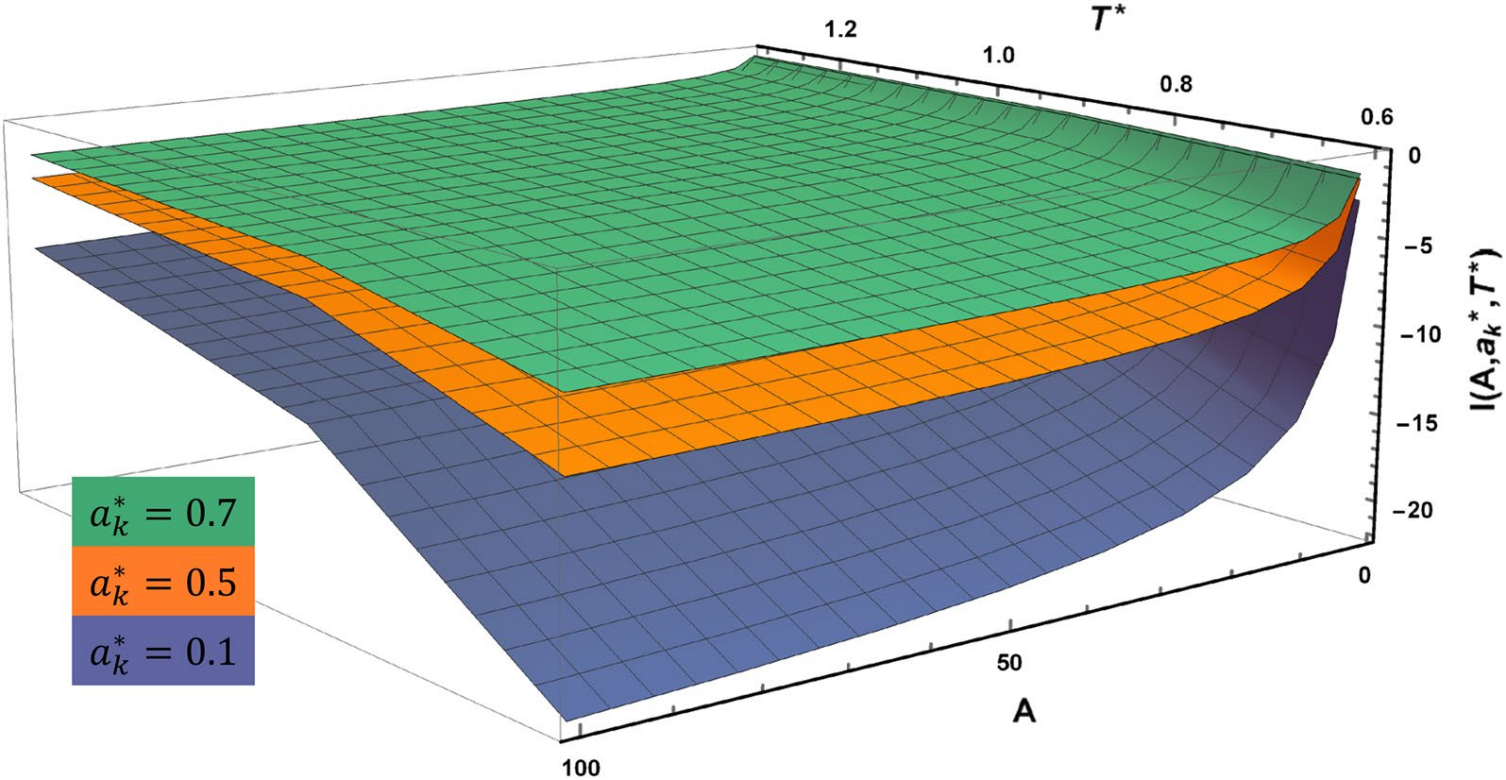
3D diagram of $I(A,{a}_{k}^{*},{T}^{*})$ versus the reduced area and temperature for different values of the reduced Kihara parameter.

The semi-analytical solution of the latter integral is as follows,

7 $$\frac{1}{V}\underset{{r}_{12}>\sigma }{\overset{ }{\iint }}\left(1-{e}^{-\beta U({r}_{12})}\right) d{V}_{1}d{V}_{2}={\sigma }_{k}^{3}\times I(A,{a}_{k}^{*},{T}^{*})$$

8 $$I\left(A,{a}_{k}^{*},{T}^{*}\right)={C}_{0}({a}_{k}^{*},{T}^{*})+\frac{{C}_{1}({a}_{k}^{*},{T}^{*})}{\sqrt{A}}+\frac{{C}_{2}({a}_{k}^{*},{T}^{*})}{A}$$

where $A={L}_{x}{L}_{y}/{\sigma }_{k}^{2}$ is the reduced area of the square cross section of the pore, ${a}_{k}^{*}=2{a}_{k}/{\sigma }_{k}$ is the reduced Kihara parameter, ${T}^{*}=T/{\varepsilon }_{k}$ is the reduced temperature, $I(A,{a}_{k}^{*},{T}^{*})$ is the numerical solution to the integral, and C_0_ to C_2_ are component- and temperature-dependent coefficients. In the L-J model, there is just one set of coefficients (C constants) used for all matters, whereas in this study, the numerical solution differs for any specific component represented by its ${a}_{k}^{*}$. Figure 4 shows the 3D diagram of $I\left(A,{a}_{k}^{*},{T}^{*}\right)$ versus $A$ and ${T}^{*}$ for three different values of ${a}_{k}^{*}$. For larger molecules, or heavier substances, ${a}_{k}^{*}$ is greater in magnitude and the changes in the value of numerical solution will be more dramatic with respect to ${T}^{*}$ or $A$. Note that Fig. 4 is the result of super time-consuming computational processes of numerical solution to the integration in Eq. (7), and is achieved with the help of Gauss–Legendre quadrature method to obtain the most possible exact outcomes.

For a bulk fluid, $A$ goes to infinity and $I(A,{a}_{k}^{*},{T}^{*})$ must be subsequently equal to C_0_. Therefore, if we take the integration of Eq. (7) from ${\sigma }_{k}$ to $\infty$, the analytical expression of C_0_ will be obtained. In this case, C_0_ and the second virial coefficient will share similar definitions and we can derive a formula for C_0_ as a function of B(T).

Based on the partition function theory, the second viral coefficient of a bulk fluid is defined as^39^

9 $$B\left(T\right)=2\pi {N}_{A}{\int }_{0}^{\infty }\left(1-{e}^{-\beta U({r}_{12})}\right) {r}^{2}dr.$$

For the very first time, we present the exact analytical solution of Eq. (9) for Kihara fluids,

10 $$\begin{gathered} B\left( {a_{k}^{*} ,T^{*} } \right) = 2\pi N_{A} \sigma_{k}^{3} \left( {1 - a_{k}^{*} } \right)^{3} \times \left( {\frac{{ - \left( {\frac{1}{{T^{*} }}} \right)^{\frac{1}{4}} }}{12\sqrt \pi }\left( {{\Gamma }\left[ { - \frac{1}{4}} \right]{\Gamma }\left[ \frac{1}{4} \right]} \right)H_{\frac{1}{2}} \left[ { - \sqrt {\frac{1}{{T^{*} }}} } \right]} \right. \hfill \\ + \frac{{3 \times a_{k}^{*} \left( {\frac{1}{{T^{*} }}} \right)^{\frac{1}{6}} }}{{\sqrt \pi \left( {1 - a_{k}^{*} } \right)}}\left( {{\Gamma }\left[ \frac{5}{6} \right]{\Gamma }\left[ \frac{4}{3} \right]} \right)H_{\frac{1}{3}} \left[ { - \sqrt {\frac{1}{{T^{*} }}} } \right] \hfill \\ \left. { + \frac{{a_{k}^{*2} \left( {\frac{1}{{T^{*} }}} \right)^{\frac{1}{12}} }}{{\sqrt \pi \left( {1 - a_{k}^{*} } \right)^{2} }}\left( {{\Gamma }\left[ {\frac{11}{{12}}} \right]{\Gamma }\left[ \frac{5}{12} \right]} \right)H_{\frac{1}{6}} \left[ { - \sqrt {\frac{1}{{T^{*} }}} } \right]} \right) \hfill \\ \end{gathered}$$

in which ${H}_{n}$ is the Hermite function of degree $n$ and $\Gamma$ is the Gamma function. Setting ${a}_{k}^{*}=0$, the last two terms in the parenthesis will be removed and we are given by the analytical formula of the second virial coefficient for the Lennard–Jones fluids. This equation is obtained with the aid of Wolfram Mathematica version 12.1^40^.

After series of manipulation (more details in Supporting Information 1), the analytical equation of C_0_ is

11 $${C}_{0}\left({a}_{k}^{*},{T}^{*}\right)=\frac{2B{\left({a}_{k}^{*},{T}^{*}\right)}}{{\sigma }_{k}^{3}\times {N}_{A}}-2{b}^{*}$$

where $${b}^{*}=\left(2\pi /3\right)\left(1-{{a}_{k}^{*}}^{3}\right)$$.

The coefficients C_1_ and C_2_ can be written as a function of C_0_ with R^2^ of 0.9997, as follows,

12 $${C}_{i}\left({a}_{k}^{*},{T}^{*}\right)={m}_{i}{C}_{0}\left({a}_{k}^{*},{T}^{*}\right)+{n}_{i} i=\mathrm{1,2}$$

in which

13 $${m}_{i}=\frac{({{\gamma }_{1})}_{i}+{\left({\gamma }_{2}\right)}_{i}\times {a}_{k}^{*}}{1+{\left({\gamma }_{3}\right)}_{i}\times {a}_{k}^{*}+{\left({\gamma }_{4}\right)}_{i}\times {{a}_{k}^{*}}^{2}}$$

14 $${n}_{i}={\left({\lambda }_{1}\right)}_{i}+{\left({\lambda }_{2}\right)}_{i}\times {a}_{k}^{*}+{\left({\lambda }_{3}\right)}_{i}\times {{a}_{k}^{*}}^{2}.$$

The constants $\gamma$ and $\lambda$ for each coefficients m and n are presented in Table 1.

**Table 1:** Values of constants $\gamma$ and $\lambda$ for m and n coefficients.

	${\gamma }_{1}$	${\gamma }_{2}$	${\gamma }_{3}$	${\gamma }_{4}$
${m}_{1}$	− 1.24981	1.757866	− 1.41068	0.193374
${m}_{2}$	0.390334	− 0.63274	− 1.67588	0.534595
	${\lambda }_{1}$	${\lambda }_{2}$		${\lambda }_{3}$
${n}_{1}$	6.559926	− 19.3229		17.89491
${n}_{2}$	− 5.89906	17.97434		− 17.1227

The Helmholtz free energy of a confined fluid is formulated by Eq. (15) obtained by applying the ideal gas free energy and considering the limited compressibility of matters via substituting $\mathrm{ln}V-\left(\frac{N}{V}\right)b=\mathrm{ln}\left(V-Nb\right)$.

15 $$F=f\left(T\right)-NkT\mathit{ln}\left(V-Nb\right)+\frac{kT{N}^{2}}{2V}{\sigma }_{k}^{3}\times I(A,{a}_{k}^{*},{T}^{*})$$

In the vdW theory, a molecule’s volume (the volume excluded from the system) is designated by the variable $b$ which represents the available space to molecules for overlap. With this definition, other molecules will have an accessible volume of $V-Nb$ to move in. This is the backbone of the idea laying behind the substitution used in Eq. (15), which is obviously independent of the applied potential function.

As Fig. 2 shows, a cross-section confinement occurs in an axially infinite pore for which the axial (${P}_{zz}$) and transverse (${P}_{xx}$ and ${P}_{yy}$) components of the pressure tensor are as follows,

16 $${p}_{ii}=-\frac{1}{V}\frac{\partial F}{\partial {\epsilon }_{ii}}$$

17 $${P}_{xx}={P}_{yy}=-\frac{{\sigma }_{k}^{2}}{L}\frac{\partial F}{\partial A} , {P}_{zz}=-\frac{{\sigma }_{k}^{2}}{A}\frac{\partial F}{\partial L}$$

Using Eqs. (17), and applying (15(, the axial and transverse components are given by Eqs. (18) and (19).

18 $${P}_{xx}={P}_{yy}=\frac{NkT}{V-Nb}-\frac{kT{N}^{2}}{{2V}^{2}}{\sigma }_{k}^{3}\left[-{C}_{0}\left({a}_{k}^{*},{T}^{*}\right)-\frac{3}{2}\frac{{C}_{1}\left({a}_{k}^{*},{T}^{*}\right)}{\sqrt{A}}-\frac{2{C}_{2}\left({a}_{k}^{*},{T}^{*}\right)}{A}\right]$$

19 $${P}_{zz}=\frac{NkT}{V-Nb}-\frac{kT{N}^{2}}{{2V}^{2}}{\sigma }_{k}^{3}\left[-{C}_{0}\left({a}_{k}^{*},{T}^{*}\right)-\frac{{C}_{1}\left({a}_{k}^{*},{T}^{*}\right)}{\sqrt{A}}-\frac{{C}_{2}\left({a}_{k}^{*},{T}^{*}\right)}{A}\right]$$

Clearly, when $A\to \infty$, the bulk vdW equation is derived and ${P}_{xx}={P}_{yy}={P}_{zz}$. In reduced coordinates, Eqs. (18 and 19) can be written as,

20 $${P}_{xx}^{*}={P}_{yy}^{*}=\frac{{T}^{*}}{{v}^{*}-{b}^{*}}-\frac{{T}^{*}}{{{v}^{*}}^{2}}\left[-\frac{{C}_{0}\left({a}_{k}^{*},{T}^{*}\right)}{2}-\frac{3}{4}\frac{{C}_{1}\left({a}_{k}^{*},{T}^{*}\right)}{\sqrt{A}}-\frac{{C}_{2}\left({a}_{k}^{*},{T}^{*}\right)}{A}\right]$$

21 $$P_{zz}^{*} = \frac{{T^{*} }}{{v^{*} - b^{*} }} - \frac{{T^{*} }}{{v^{*2} }}\left[ {\underbrace {{ - \frac{{C_{0} \left( {a_{k}^{*} ,T^{*} } \right)}}{2} - \frac{1}{2}\left( {\frac{{C_{1} \left( {a_{k}^{*} ,T^{*} } \right)}}{\sqrt A } + \frac{{C_{2} \left( {a_{k}^{*} ,T^{*} } \right)}}{A}} \right)}}_{{ - {\varvec{I}}\left( {{\varvec{A}},{\varvec{a}}_{{\varvec{k}}}^{*} ,{\varvec{T}}^{*} } \right)/2}}} \right]$$

where ${P}^{*}=P{\sigma }_{k}^{3}/\varepsilon \left[Pa.{m}^{3}.{J}^{-1}\right]$; ${T}^{*}=kT/\varepsilon \left[J.{K}^{-1}.K.{J}^{-1}\right]$; ${v}^{*}=\left(V/N\right){\sigma }_{k}^{-3} \left[{m}^{3}.{m}^{-3}\right]$; and ${b}^{*}=b{\sigma }_{k}^{-3} \left[{m}^{3}.{m}^{-3}\right]$. Equations (20 and 21) have a format akin to the standard vdW cubic EOS, but we exclusively observe that the attractive term is a function of temperature, and this would not be possible except with the exact solution of Eq. (6).

From the theory of corresponding states and the continuity principle of gaseous and liquid phases, the following conditions are required for vdW type EOSs at the critical point^41^:

22 $${\left(\frac{\partial {P}_{zz}^{*}}{\partial {v}^{*}}\right)}_{{T}^{*}={T}_{c}^{*}}={\left(\frac{{\partial }^{2}{P}_{zz}^{*}}{\partial {{v}^{*}}^{2}}\right)}_{{T}^{*}={T}_{c}^{*}}=0$$

Applying the requirements on Eq. (21), we can establish the following relations with which the critical properties of a Kihara fluid can be obtained:

23 $${\varvec{I}}({\varvec{A}},{{\varvec{a}}}_{{\varvec{k}}}^{\boldsymbol{*}},{{\varvec{T}}}_{{\varvec{c}}}^{\boldsymbol{*}})={C}_{0}\left({a}_{k}^{*},{T}_{c}^{*}\right)+\frac{{C}_{1}({a}_{k}^{*},{T}_{c}^{*})}{\sqrt{A}}+\frac{{C}_{2}({a}_{k}^{*},{T}_{c}^{*})}{A}=-\frac{27}{4}{{\varvec{b}}}^{\boldsymbol{*}}$$

24 $${P}_{c}^{*}=\frac{{T}_{c}^{*}}{8{b}^{*}}$$

25 $${v}_{c}^{*}=3{b}^{*}$$

Not only Eqs. (23 and 24) reveal a shift in critical temperature and pressure of fluids, but they imply that in spite of the L-J critical shift equations^6^, it is not feasible to directly calculate the critical temperature. Herein the focus is on the critical temperature shift of substances.

At the critical point and based on Eq. (21), the EOS will possess the following format:

26 $${P}_{c}^{*}=\frac{{T}_{c}^{*}}{{v}_{c}^{*}-{b}^{*}}-\frac{{T}_{c}^{*}\left(-I\left(A,{T}_{c}^{*},{a}_{k}^{*}\right)/2\right)}{{{v}_{c}^{*}}^{2}}$$

Rearranging the relation and replacing ${v}_{c}^{*}={Z}_{c}{T}_{c}^{*}/{P}_{c}^{*}$, we get the below cubic relation,

27 $${Z}_{c}^{3}-\left(1+\frac{{P}_{c}^{*}}{{T}_{c}^{*}}{b}^{*}\right){Z}_{c}^{2}+\frac{{P}_{c}^{*}}{{T}_{c}^{*}}\left(-\frac{I\left(A,{T}_{c}^{*},{a}_{k}^{*}\right)}{2}\right){Z}_{c}-\frac{{{P}_{c}^{*}}^{2}}{{{T}_{c}^{*}}^{2}}\left(-\frac{I\left(A,{T}_{c}^{*},{a}_{k}^{*}\right)}{2}\right){b}^{*}=0$$

which is the original vdW EOS provided we substitute ${b}^{*}={T}_{c}^{*}/8{P}_{c}^{*}$ and $I\left(A,{T}_{c}^{*},{a}_{k}^{*}\right)=\left(-27/4\right){b}^{*}$. Therefore, regardless of pore size and the critical shift effect or the presence of temperature in the attractive term, Eq. (27) has always the exact single root of vdW, i.e., 3/8.

**Figure 5:**
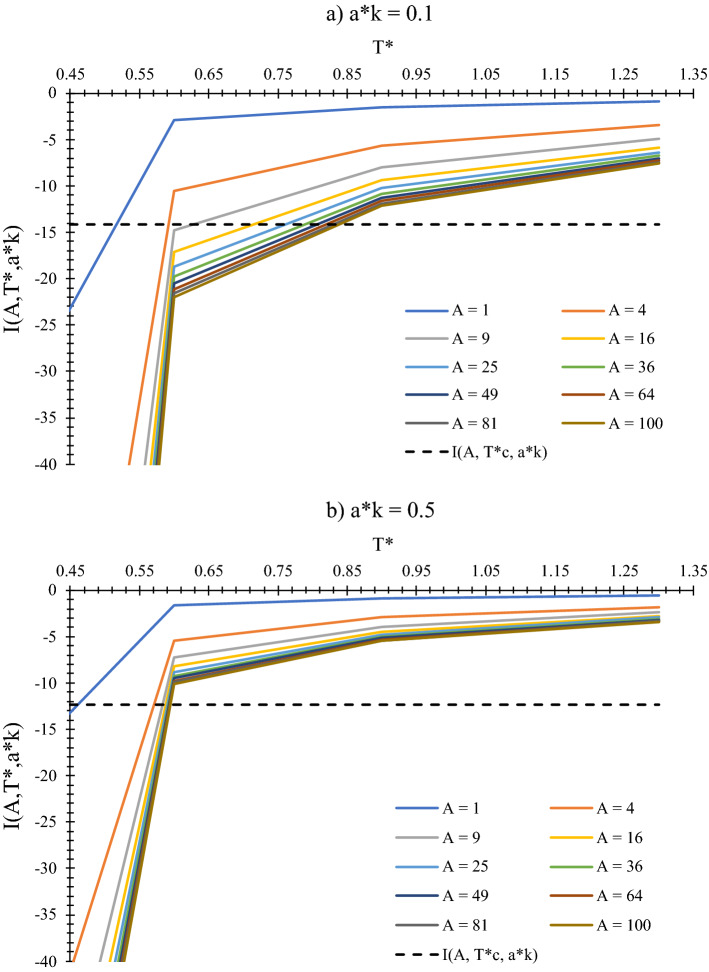
Diagram of the integral numerical solution, $I(A,{T}^{*},{a}_{k}^{*}$), versus ${T}^{*}$ for two types of fluids; (**a**) ${a}_{k}^{*}=0.1$ representing light-weight molecules, and (**b**) ${a}_{k}^{*}=0.5$ representing mid-weight molecules. Dashed line denotes the value of the numerical integration at the critical point, $I(A,{T}_{c}^{*},{a}_{k}^{*}$), and its intersections with diagram curves determine the value of ${T}_{c}^{*}$ at each pore size.

Being the focal point of this study, Eq. (23) hints that the numerical solution to Eq. (7) at the critical point – $I(A,{a}_{k}^{*},{T}_{c}^{*})$ – always owns a constant value of $-27/4\times {b}^{*}$, which is independent of the critical temperature or pore area ($A$), and is only a function of fluid type (${a}_{k}^{*}$). For a detailed inspection, Fig. 5 provides a representation of the essence of Eq. (23) where Fig. 4 and the term $-27/4\times {b}^{*}$ come together, as identified by the black dashed line. Their intersections represents the values of ${T}_{c}^{*}$ at various pore radii which levels out sooner for larger molecules (Fig. 5b) than the lighter ones (Fig. 5a).

**Figure 6:**
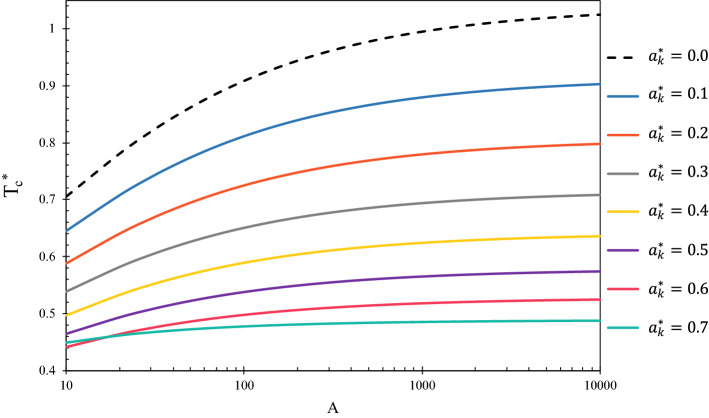
Value of ${T}_{c}^{*}$ versus pore size for different values of ${a}_{k}^{*}$ (different fluids).

As discussed earlier, Eq. (23) must be solved numerically to find the critical temperature at the desired pore radius using the Kihara parameters gained from fluid’s properties^42^. Figure 6 demonstrates the dimensionless diagram of numerically calculated values of ${T}_{c}^{*}$ for a limited range of dimensionless parameters $A$ and ${a}_{k}^{*}$. Yet, in the L-J model, ${T}_{c}^{*}$ merely depends on $A$ (or pore size) and the effect of fluid type is not included^43^.

To calculate the critical temperature of a confined fluid, whether of Kihara or L–J type, one should have ${\sigma }_{k}$, ${a}_{k}$, ${r}_{p}$, and ${\varepsilon }_{k}$ known. Then ${a}_{k}^{*}$ and $A$ are given to Eq. (23) as inputs and ${T}_{c}^{*}$ will be obtained after rooting find. Alternatively, Fig. 6 is available as a dimensionless chart and can be employed for light and semi-heavy molecules as well as the L–J type fluids within the nanometric pore sizes. Note that, in this figure, the ${a}_{k}^{*}=0$ curve represents the L–J fluid type, if the exact solution procedure is followed with the Lennard–Jones potential function.

**Figure 7:**
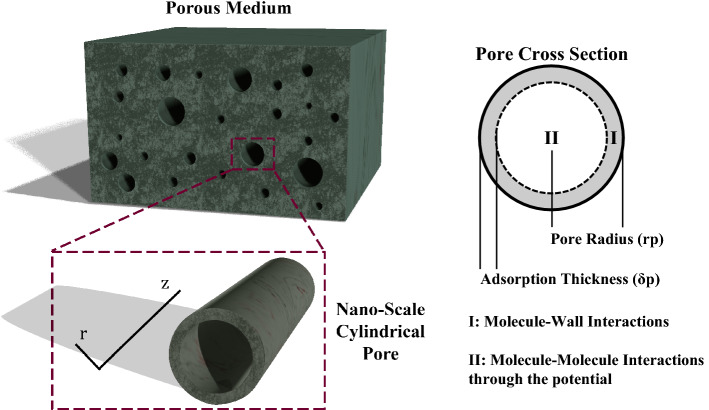
Schematic of a nano-scale pore in cylindrical coordinates including the adsorption thickness effect.

It should be noted that all the aforementioned calculations can correspondingly be applied to nano-scale pores in cylindrical coordinates (Fig. 7) where pores are infinite over the Z direction and $A=\pi {\left({r}_{p}/{\sigma }_{k}\right)}^{2}$.

**Figure 8:**
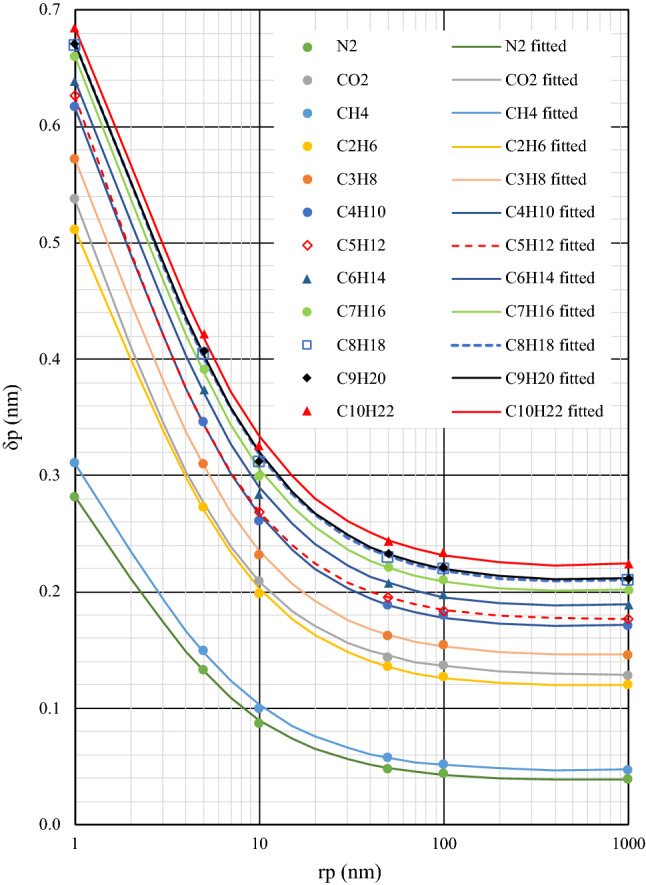
Measured 23 and fitted values of adsorption thickness.

The molecule-wall interactions cannot be ignored in molecular-scale studies; such interactions lead to a layer of fluid’s molecules stuck on the surface of pore walls, known as adsorption thickness (Fig. 7). In this regard, there will be a slight change to the definition of dimensionless area (A) as follows:

28 $$A=\pi {\left(\frac{{r}_{p}-{\delta }_{p}}{{\sigma }_{k}}\right)}^{2}$$

where ${\delta }_{p}$ is the adsorption thickness for which here we employ the values Zhang et al.^23^ have reported in their work. In this study, instead of a logarithmic function proposed in their paper, a rational model as Eq. (29) is used for least square fitting that results in less deviation:

29 $${\delta }_{p}=\frac{a+b{\times r}_{p}}{1+c\times {r}_{p}+d\times {r}_{p}^{2}}$$

in which ${r}_{p}$ and ${\delta }_{p}$ are both in nm. Moreover, a, b, c, and d are coefficients whose values are listed in Table 2 for N2, CO2, and normal hydrocarbons from C1 to C10. Figure 8 shows the measured^23^ and fitted values of ${\delta }_{p}$. Furthermore, the following expressions may be used for normal alkanes based on the values presented in Table 2:

30 $$a = 0.12 \ln \omega + 1.0495 {\text{R}}^{2} = 0.9235$$

31 $$b = 0.0225 \ln \omega + 0.1332 {\text{R}}^{2} = 0.8675$$

32 $$c = - 1.0654 \omega^{2} + 0.1643 \omega + 0.6555 {\text{R}}^{2} = 0.7315$$

33 $$d = 6.3464 \times 10^{ - 6} \ln \omega - 5.9508 \times 10^{ - 6} {\text{R}}^{2} = 0.8454$$

**Table 2:** Values of coefficients in the adsorption thickness equation (Eq. 29).

	N2	CO2	C1	C2	C3	C4	C5	C6	C7	C8	C9	C10
$a$	0.4441	0.8646	0.4848	0.7678	0.8601	0.9029	0.9543	0.8985	0.9178	0.9136	0.9139	0.9218
$b$	0.0239	0.1009	0.0288	0.0748	0.0949	0.1051	0.1257	0.1033	0.1080	0.1062	0.1071	0.1096
$c$	0.6594	0.7955	0.6520	0.6465	0.6687	0.6338	0.7231	0.5655	0.5526	0.5209	0.5203	0.5048
$d (\times 1{0}^{-5})$	− 3.849	− 0.435	− 3.695	− 1.809	− 1.462	− 1.620	− 0.867	− 1.535	− 1.422	− 1.371	− 1.143	− 1.393
${R}^{2}$	0.9997	0.9999	0.9996	0.9997	0.9998	0.9997	0.9999	0.9996	0.9996	0.9996	0.9995	0.9995

Considering the wall-molecule interactions, the critical shift phenomena is now modeled better and more realistic than the previous work^6^ which was developed for neutral pore walls.

### Adjustment: the bulk critical conditions must be met in ${\varvec{A}}=\boldsymbol{\infty }$

As we discussed earlier, Eq. (23) is the key formula of this research. For any given values of Kihara parameters, this formula provides the critical temperature of a fluid under confinement at any desired pore radius. The problem is, however, that potential function parameters are derived through thermodynamic data fitting of different sources. Therefore, a calculated set of parameters from one data set, e.g. $B(T)$, may (and will) differ from those of another (e.g. $\mu$). This occurs because potential functions are merely a representative and approximation of the real potential behavior of two interactive molecules. Only for a true potential there is one unique set of parameters obtained from different properties of the same fluid^20^.

Inserting any precalculated Kihara (or L–J) parameters^21,42,44,45^ in Eq. (23), the bulk critical temperature (${T}_{c}$) (and subsequently the bulk critical pressure (${P}_{c}$) from Eq. (24)) is expected to be achieved when $A$ approaches infinity: $I\left(A,{a}_{k}^{*},{T}_{c}^{*}\right)={C}_{0}\left({a}_{k}^{*},{T}_{c}^{*}\right)$. Nevertheless, there is a remarkable deviation for both Kihara and L-J potential functions when calculating bulk ${T}_{c}$, as listed in Table 3.

**Table 3:** Values of calculate bulk critical temperatures from Eq. (23) based on different literature reports of Kihara and L-J parameters for some fluids.

	Kihara				Lennard–Jones			
	Set 1^42^		Set 2^45^		Set 1^21^		Set 2^44^	
	${T}_{c}[K]$	Error %	${T}_{c}[K]$	Error %	${T}_{c}[K]$	Error %	${T}_{c}[K]$	Error %
CH_4_	178.30	6.43	171.56	9.97	148.40	22.12	153.93	19.22
C_2_H_6_	369.76	21.11	–	–	201.65	33.95	252.40	17.33
n-C_4_H_10_	446.84	5.11	–	–	232.40	45.33	308.45	27.44
n-C_5_H_12_	504.52	7.41	504.58	7.43	226.11	51.86	–	–
CO_2_	275.54	9.42	–	–	199.69	34.35	196.31	35.46
Ar	132.56	12.04	132.63	11.99	122.70	18.58	124.43	17.43

Hence, the model must be somehow **adjusted** in that regard. An adjustment method for the Lennard–Jones potential function was presented by Zarragoicoechea and Kuz^43^ applying a rough approximation to Eq. (6). While broadly drawing the attention of researchers, that adjustment method seems to be a numerical correlation and lacks a thermodynamic basis. Here we propose a new adjustment procedure with which the mentioned bulk state conditions will be satisfied with a thermodynamic basis, and there will be no need for auxiliary equations. To this end, the potential function’s parameters will be manipulated until Eqs. (23 and 24) result in the critical properties of a fluid in the macroscopic state.

Approaching the cross section to infinity and rewriting Eqs. (23 and 24), we have (more details in Supporting Information 2):

34 $$\frac{2}{{N}_{A}}{\left.B\left({a}_{k}^{*},T/{\varepsilon }_{k}\right)\right|}_{T={T}_{C}}=-\frac{19}{32}\times \frac{k{T}_{c}}{{P}_{C}}$$

35 $$\frac{2\pi }{3}\left({\sigma }^{3}-{\left(2{a}_{k}\right)}^{3}\right)=\frac{1}{8}\times \frac{k{T}_{c}}{{P}_{C}}$$

where ${T}_{c}$ and ${P}_{c}$ are in K and Pa, respectively.

#### Adjustment for Lennard–Jones potential function

For a two-parameter potential like Lennard–Jones, Eqs. (34 and 35) suffice and must be solved to compute ${\sigma }_{LJ}$ and ${\left({\varepsilon }_{k}\right)}_{LJ}$. Table 4 lists the values of adjusted L–J parameters for nitrogen, carbon dioxide, and a wide range of common hydrocarbons used in the literature. Equations 36 and 37 declare that ${T}_{c}^{*}$ and ${P}_{c}^{*}$ are constant for all components in Table 4 providing we substitute the values.

**Table 4:** Adjusted L-J parameters of nitrogen, carbon dioxide, and light to semi-heavy normal alkanes.

Component	${\sigma }_{LJ}$ [Å]	${\left({\varepsilon }_{k}\right)}_{LJ} \left[K\right]$
N_2_	3.128	121.40
CO_2_	3.238	292.86
CH_4_	3.244	183.46
C_2_H_6_	3.724	293.95
C_3_H_8_	4.155	356.06
C_4_H_10_	4.519	409.29
C_5_H_12_	4.861	452.21
C_6_H_14_	5.171	488.70
C_7_H_16_	5.456	520.08
C_8_H_18_	5.731	547.52
C_9_H_20_	5.980	572.17
C_10_H_22_	6.225	594.69
C_11_H_24_	6.465	615.20
C_12_H_26_	6.679	633.49

36 $${\left(1/{T}_{c}^{*}\right)}_{LJ}=0.96275$$

37 $${\left(1/{P}_{c}^{*}\right)}_{LJ}=16.1313$$

On the other hand, ${v}_{c}^{*}$ was constant in the first place (${v}_{c}^{*}=3{b}^{*}$) and is equal to $2\pi$ for L–J fluids. Accordingly, we have,

38 $${Z}_{c}=\frac{{v}_{c}^{*}{P}_{c}^{*}}{{T}_{c}^{*}}=\frac{2\pi \times 0.96275}{16.1313}=\frac{3}{8}$$

which strongly supports the calculations of parameters and the hypotheses behind the approach. With this format, the macroscopic and microscopic theories of corresponding states are neatly related to each other, aligned with the literature established correlations^20^.

#### Adjustment for Kihara potential function

A three-parameter potential function such as Kihara, however, requires another relation (for ${a}_{k}$). This third equation will be the definition of the reduced Kihara size parameter as follows:

39 $${a}_{k}^{*}=\frac{2{a}_{k}}{{\sigma }_{k}}$$

Although Kihara parameters have different values regarding the property they have been fitted with, the ratio of the two size parameters ${a}_{k}$ and ${\sigma }_{k}$ as ${a}_{k}^{*}$ appears to be almost **constant** 42. Thus, we need the value of ${a}_{k}^{*}$ as the final step. In this study, we will exclusively obtain ${a}_{k}^{*}$ through fitting the Kihara parameters with the DIPPR reports of $B(T)$, using Eq. (10). Values of ${a}_{k}^{*}$ determined in this work are brought in Table 5, and Eq. (40) can be used for n-alkanes with R^2^ of 0.98. Complete details are presented in Supporting Information 1 and 2.

**Table 5:** Value of ${a}_{k}^{*}$ obtained from the second virial coefficient data fitting.

Component	N_2_	CO_2_	CH_4_	C_2_H_6_	C_3_H_8_	C_4_H_10_	C_5_H_12_
${a}_{k}^{*}=2{a}_{k}/{\sigma }_{k}$	0.1666	0.4837	0.1456	0.2389	0.3678	0.4872	0.5718
Component	C_6_H_14_	C_7_H_16_	C_8_H_18_	C_9_H_20_	C_10_H_22_	C_11_H_24_	C_12_H_26_
${a}_{k}^{*}=2{a}_{k}/{\sigma }_{k}$	0.6000	0.6980	0.6678	0.7389	0.7772	0.8147	0.8767

40 $${a}_{k}^{*}=0.219{\left(\omega \times MW\right)}^{0.3013}$$

With ${a}_{k}^{*}$ in hand, we can use the adjustment method (Eqs. 34, 35, and 39) to calculate the adjusted Kihara parameters by which the critical conditions of a bulk fluid will be met. Their values are listed for different substances in Table 6, and Eqs. (41 and 42) are the fitted relations for ${\varepsilon }_{k}$ and ${\sigma }_{k}$ with R^2^ of 0.990 and 0.999, respectively.

**Table 6:** Adjusted Kihara parameters of nitrogen, carbon dioxide, and light to semi-heavy normal alkanes.

Component	${a}_{k}$ [Å]	${\sigma }_{k}$ [Å]	${\varepsilon }_{k} \left[K\right]$
N_2_	0.2610	3.1332	150.16
CO_2_	0.8152	3.3705	523.47
CH_4_	0.2364	3.2475	220.96
C_2_H_6_	0.4469	3.7409	397.73
C_3_H_8_	0.7772	4.2264	560.99
C_4_H_10_	1.1468	4.7079	734.18
C_5_H_12_	1.4889	5.2079	882.85
C_6_H_14_	1.6823	5.6079	980.08
C_7_H_16_	2.1871	6.2671	1139.2
C_8_H_18_	2.1529	6.4475	1168.2
C_9_H_20_	2.6244	7.1036	1297.4
C_10_H_22_	2.9881	7.6895	1391.1
C_11_H_24_	3.4135	8.3795	1482.1
C_12_H_26_	4.2524	9.7014	1598.5

41 $${\left(\frac{1}{{T}_{c}^{*}}\right)}_{Kihara}=1.69024{a}_{k}^{*}+0.925$$

42 $${\left(\frac{1}{{P}_{c}^{*}}\right)}_{kihara}=-25.095{{a}_{k}^{*}}^{4}-32.278{{a}_{k}^{*}}^{3}+22.037{{a}_{k}^{*}}^{2}+19.008{a}_{k}^{*}+16.248$$

Even though ${T}_{c}^{*}$ and ${P}_{c}^{*}$ are component-dependent in the Kihara model, the critical molar volume has the same behavior – ${v}_{c}^{*}=2\pi \left(1-{{a}_{k}^{*}}^{3}\right)$ – to reflect the fact that ${Z}_{c}$ is constant (and equal to 3/8) for all vdW fluids regardless of the potential function. This can be simply checked through assuming an arbitrary value for ${a}_{k}^{*}$ and calculating ${Z}_{c}$, as we did for L–J in Eq. (38).

As we perceived, ${T}_{c}^{*}$ and ${P}_{c}^{*}$ was constant for all matters following the L–J model. Since the Kihara potential somehow encompasses the L–J function in its formula, the intercepts of Eqs. (41 and 42) are expected to be equal to their corresponding values in Eqs. (36 and 37). Although they are not exactly the same, they are sufficiently close and the deviation can be attributed to the uncertainties in data employed for ${T}_{c}$ and ${P}_{c}$ ^41^, fitting issues, or imperfect root finding. Thus, the corresponding states principle of Kihara fluids is indeed the generalized form of that of the Lennard–Jones model.

### Variable energy parameter (ε)

Thus far, (1) the exact solution of the free energy integral was presented using the Kihara potential function and the critical temperature shift equation was derived (Section "How to better model the critical temperature of confined fluids?"), (2) a new thermodynamic-based adjustment method was employed, (3) the Kihara parameters were fitted using the DIPPR database of $B(T)$, and (4) the adjusted Kihara and L–J parameters were listed for nitrogen, carbon dioxide, and normal alkanes from C1 to C12 (Section "Adjustment: the bulk critical conditions must be met in ").

**Figure 9:**
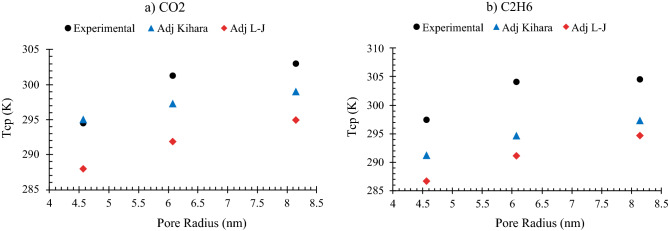
Comparison among the T_cp_ values from experimental reports 4 as well as the predictions of adjusted Kihara and adjusted L-J methods for (**a**) carbon dioxide and (**b**) ethane.

The adjustment satisfied the critical point requirements of macroscopic fluids, but it does not necessarily enable the model to provide better predictions of pore critical temperature (T_cp_). As shown for CO_2_ and C_2_H_6_ in Fig. 9, for both Kihara and L–J potentials, ${T}_{cp}$ values predicted by the adjustment method deviate noticeably from the experimental values 4. This implicitly implies that the confinement effect is witnessed in larger pore sizes in the Lennard–Jones model, sooner than that of the Kihara, and both earlier than the empirical observations. Such imperfection in the calculations, however, is happening while the free energy integral is solved exactly (making the EOS entirely exclusive for each component) and the adjustment is implemented to fulfill the bulk conditions. Therefore, the theory needs a moderator to alleviate the pore-scale problem.

Let us tackle the issue from a new and different perspective. The critical point is known as the characteristic state, where the liquid and gaseous phases of a substance become identical. At this point, the critical temperature is a measure of kinetic energy, therefore a relation between ${\varepsilon }_{k}$ and ${T}_{c}$ is quite expectable and reasonable^20^:

43 $$\frac{\varepsilon }{k}={\varepsilon }_{k}={A}_{1}{T}_{c}$$

where ${A}_{1}$ is a universal constant obtained by quantum mechanics studies. It is experimentally proved and measured for simple fluids (with spherical molecules) obeying the L–J potential function that ${A}_{1}$ is approximately equal to 0.77^44^. Please refer to Supporting Information – Molecular Theory of Corresponding States for more explanations.

From the foregoing explanations, knowing that ${T}_{c}$ and ${\varepsilon }_{k}$ are of one type, and based on the shift of critical temperature of confined fluids, we can **infer** that the energy parameter of the potential function would accordingly shift. In other words, as a component has different critical temperature values at different pore radii, there might be also a possibility for ${\varepsilon }_{k}$ to vary in pore size–which is designated herein as ${\varepsilon }_{kp}$. In fact, since tuning has always been prevalent among PVT researchers to accomplish their desired outcomes, we are considering ${\varepsilon }_{k}$ a **tuning parameter** that decreases as pore size shrinks.

So why manipulating $${\varepsilon }_{k}$$ rather than $${\sigma }_{k}$$ or $${a}_{k}$$? Along the lines of $${\varepsilon }_{k}$$ and $${T}_{c}$$, there is a relation between the size parameters $${a}_{k}$$ or $${\sigma }_{k}$$ and the critical molar volume ($${v}_{c})$$, because the latter represents the molecular size. The point is, however, that $${v}_{c}$$ of a bulk fluid does not really differ from the confined one. Not only this claim is theoretically conspicuous from Eq. (25) where there is no confinement term (no sign of $$A$$), but experiments have also made the same assertion as well^43^. Even if there were any shift of $${v}_{c}$$ regarding pore shrinking, it would be irrelevant to choose a distance parameter for calibrating an energy parameter ($${T}_{c}$$). Thus, $${\varepsilon }_{k}$$ (or in fact $$\varepsilon$$) seems to be the only choice for manipulation. Meanwhile, $${a}_{k}$$ and $${\sigma }_{k}$$ must be involved in the calculations as they have been determined from the adjustment process.

To be more specific, such an inference can be conceptually paraphrased as if the parameter ${\varepsilon }_{k}$ in the original integral (Eq. 7) is a function of the pore area (A) over which the integration is taken, i.e., Eq. (44). However, the solution to the integral, written as Eq. (45), does not fundamentally differ from the original solution (Eq. 8), except ${\varepsilon }_{k}$ must be replaced by ${\varepsilon }_{kp}$.

44 $$\frac{1}{V}\underset{A}{\overset{ }{\int }}\left(1-\mathit{exp}\left[-\frac{4}{T}{\varepsilon }_{k}\left[A\right]\left({\left(\frac{{\sigma }_{k}-2{a}_{k}}{r-2{a}_{k}}\right)}^{12}-{\left(\frac{{\sigma }_{k}-2{a}_{k}}{r-2{a}_{k}}\right)}^{6}\right)\right]\right)dr$$

45 $$I\left(A,{a}_{k}^{*},T/{\varepsilon }_{k}\left[A\right]\right)={C}_{0}({a}_{k}^{*},T/{\varepsilon }_{k}\left[A\right])+\frac{{C}_{1}({a}_{k}^{*},T/{\varepsilon }_{k}\left[A\right])}{\sqrt{A}}+\frac{{C}_{2}({a}_{k}^{*},T/{\varepsilon }_{k}\left[A\right])}{A}$$

We deliberately write ${\varepsilon }_{k}[A]$ instead of ${\varepsilon }_{k}[{r}_{p}]$ to avoid ambiguity. Indeed, $r$ (intermolecular distance) is totally different from ${r}_{p}$ which is the maximum distance that two molecules can be placed far from each other, and almost equates the pore diameter (${r}_{max}\cong 2{r}_{p}$). Substituting ${\varepsilon }_{k}\left[A\right]$ for ${\varepsilon }_{k}$, we still should refer to Eq. (23) to calculate the pore critical temperature, because, the variable energy parameter has no effect on the procedure with which Eq. (23) is obtained, nor on the dimensionless diagrams.

How to determine the value of ${\varepsilon }_{kp}$? Presume that universally unique and reliable experimental measurements of critical temperature of confined pure fluids are available. In other words, the present $({r}_{p},{T}_{cp}$) data points are real, certain, and precise. In this case, the corresponding ${\varepsilon }_{kp}$ will be obtained if we substitute the known properties of the component (${\sigma }_{k}$, ${a}_{k}^{*}$, ${r}_{p}$, ${T}_{cp}$) into Eq. (23). With a reverse rooting find try, ${\varepsilon }_{kp}$ will be accurately determined as the unknown of the equation. Nonetheless, literature reports of ${T}_{cp}$, whether empirically measured or numerically simulated, are highly uncertain and scattered. Therefore, we resort to calculating ${\varepsilon }_{kp}$ for each available pair of $({r}_{p}$, ${T}_{cp}$) from any reference, and will propose a model that fits best, if possible. We state “if possible”, because on occasion, the model is not able to give an ${\varepsilon }_{kp}$ less than the bulk ${\varepsilon }_{k}$ (${\varepsilon }_{kb}$) for some matters such as CO_2_, or there is too small number of data points to fit a model, like C_3_H_8_.

Similar to the fact that ${T}_{cp}$ equates ${T}_{c}$ when ${r}_{p}$ increases and approaches infinity, ${\varepsilon }_{kp}$ must behave the same towards ${\varepsilon }_{kb}$ (i.e. ${\varepsilon }_{k}$ from adjustment). Thus, the fitting model would possess a format as below,

46 $${\varepsilon }_{kp}={\varepsilon }_{kb}\left(1-\frac{\alpha }{{A}^{\beta }}\right)$$

where $A=\pi {\left(\left({r}_{p}-{\delta }_{p}\right)/{\sigma }_{k}\right)}^{2}$, $\alpha$ and $\beta$ are component-dependent fitting constants.

**Figure 10:**
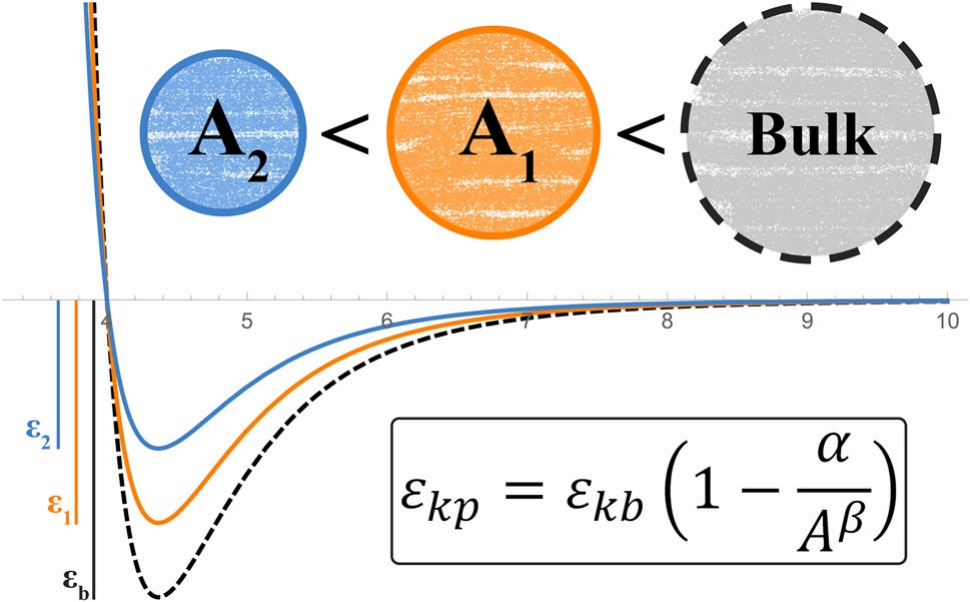
Depiction of pore-dependent energy parameter.

Figure 10 summarizes the notion of pore-size-dependent energy parameter and depicts how the potential curve changes as a result. Although changing ${\varepsilon }_{k}$ appears to be merely a tuning attitude that we have adopted, we deeply look forward to knowing whether the confinement phenomenon truly affects the ultimate and real intermolecular interactions. Yet, molecular-scale experimental measurements are extremely challenging to perform and are accompanied by uncertainties.

## Results and discussion

### Calculating the values of ${{\varvec{\varepsilon}}}_{{\varvec{k}}{\varvec{p}}}$ (Training Section)

For the very first time, we have gathered 76 data points of pore critical temperature reported in the literature, the largest collection ever employed. This collection covers 9 components including methane, ethane, propane, n-butane, n-pentane, n-octane, n-decane, nitrogen, and carbon dioxide, from 13 different references overall^1–4,9,11,13–17^. Values of ${\varepsilon }_{kp}$ (Kihara) are calculated and categorized with respect to components and references, and are listed in Table 7. This classification helps readers to conveniently select their desired ${\varepsilon }_{kp}$ based on their and the reference’s hypotheses.

**Table 7:** Calculated values of ${\varepsilon }_{kp}$ for each component based on different sources of ${T}_{cp}$ reports along with their fitting coefficients.

Ref	CH_4_			C_2_H_6_			C_4_H_10_			C_5_H_12_			C_8_H_18_			C_10_H_22_			N_2_			CO_2_		
	${r}_{p}$	${T}_{cp}$	${\varepsilon }_{kp}$	${r}_{p}$	${T}_{cp}$	${\varepsilon }_{kp}$	${r}_{p}$	${T}_{cp}$	${\varepsilon }_{kp}$	${r}_{p}$	${T}_{cp}$	${\varepsilon }_{kp}$	${r}_{p}$	${T}_{cp}$	${\varepsilon }_{kp}$	${r}_{p}$	${T}_{cp}$	${\varepsilon }_{kp}$	${r}_{p}$	${T}_{cp}$	${\varepsilon }_{kp}$	${r}_{p}$	${T}_{cp}$	${\varepsilon }_{kp}$
	nm	K	K	nm	K	K	nm	K	K	nm	K	K	nm	K	K	nm	K	K	nm	K	K	nm	K	K
4				4.57	297.5	406.3																4.57	294.5	522.5
				6.08	304.1	410.5																6.08	301.3	530.6
				8.15	304.6	407.5																8.15	303.0	530.5
13																								
Mica	4.22	182.3	222.2				4.28	397.5	718.3				5.54	530.4	1116									
	3.21	176.6	218.6				3.26	382.7	701.5				4.41	516.0	1093									
	2.69	171.3	214.7				2.71	374.6	694.9				3.30	503.8	1079									
	2.15	164.7	210.4				2.17	359.3	678.3				2.20	466.9	1022									
	1.63	152.4	200.6				1.64	331.2	643.9				1.66	429.4	960.9									
13																								
Graphite	4.14	183.4	223.7				4.17	402.1	727.5				5.28	540.6	1139									
	3.15	177.9	220.5				3.19	389.8	715.4				4.25	531.7	1128									
	2.61	174.3	219.0				2.68	379.0	703.7				3.22	513.9	1101									
	2.11	169.0	216.3				2.15	361.6	683.4				2.17	470.7	1031									
	1.62	152.2	200.7				1.63	324.2	630.5				1.65	416.6	932.8									
17	2	155.2	199.8	2	248.7	360.7				2	337.3	693.7				2	475.9	749.1						
	3	172.7	214.8	3	273.7	383.1				3	375.7	749.9				3	542.4	928.6						
	5	179.8	217.5	5	282.4	384.1				5	396.4	772.4				5	575.6	1076						
				7	286.8	385.4				6	399.7	774.1												
1,2,3																			1.2	87	121.8			
																			1.9	98.12	129.5			
																			2.2	103	134.1			
																			3.0	105	133.5			
																			3.2	105	133.0			
																			3.9	112	140.3			
9	2	158.1	203.4																					
	3	175.8	218.5																					
	5	183.4	221.8																					
14	7	183.3	219.0	7	292	392.3																		
	10	186.0	220.3	10	296.3	394.4																		
15				3	258	361.1																		
				4	281.5	387.0																		
				5	292	397.2																		
				6	298	402.4																		
				7	300	403.1																		
				8	302	404.2																		
				9	304	405.6																**C**_**3**_**H**_**8**_		
16				5	285.1	387.7																${r}_{p}$	${T}_{cp}$	${\varepsilon }_{kp}$
				6	292.5	395.0																nm	K	K
11	2.03	169.7	218.0	2.03	269.5	390.2	2.03	372.0	706.8													2.03	320.7	538.4
$\boldsymbol{\alpha }$	74.963			5.8639			7.0572			0.8501			1.7739			1.5252			0.6989			Not Available		
${\varvec{\beta}}$	1.5236			0.9898			1.1075			0.2694			0.7408			0.3919			0.3443			Not Available		
${R}^{2}$	0.75				0.40		0.86			0.93			0.96			0.99			0.87			Not Available		

Methane and ethane had the greatest number of available ${T}_{cp}$ reports for training, i.e. 19, though the scattering of ethane points is much more than that of methane, which is readily notable from their R^2^. As discussed earlier, the obtained ${\varepsilon }_{kp}$ from our theory had a greater value than ${\varepsilon }_{kb}$ for a few numbers of (${r}_{p}$,${T}_{cp}$) points. On the one hand, this is because the points would need a flexibility beyond what the model is capable of. On the other hand, there is always an amount of uncertainty in both numerical simulations and empirical measurements to which such an imperfection could be assigned. That is why it was impossible to fit a model for CO_2_ whose two ${\varepsilon }_{kp}$ values out of the three, were bigger than its bulk ${\varepsilon }_{k}$.

Equations (47 and 48) can be respectively used for the values of $\alpha$ and $\beta$ of Table 7, with R^2^ of 0.99 and 0.96.

47 $$\alpha =(-50.8878+459.659\times {a}_{k}^{*})/(-15.8293+MW+{\omega }^{2})$$

48 $$\beta =1.76384-2.11073/\sqrt{\alpha }+0.74907/\alpha$$

The Lennard–Jones potential function is not an appropriate choice of changing ${\varepsilon }_{kp}$. Although it is feasible to determine ${\left({\varepsilon }_{kp}\right)}_{LJ}$ for each (${r}_{p}$,${T}_{cp}$) point, the insufficient flexibility of this potential forbids having valid values; for the Kihara model, only 12 data points (16% out of 76) had an ${\varepsilon }_{kp}$ greater than ${\varepsilon }_{kb}$. In opposite, L-J suffers more from this problem for which there were 37 (almost 50%) theoretically-false results (See Supporting Information.xlsx). It is directly related to the curvature of $U({r}_{12})$ and the fact that mostly the predictions provided by the L–J model are underestimated. Therefore, the Kihara potential function is, again, a more decent choice to go for.

### Solid verification of the proposed model (testing section)

In this section, we assess the performance of our model with another 18 data points of recently published critical shift reports. These would include measured T_cp_ and P_cp_ values of methane^46^, and Tcp reports of carbon dioxide and normal heptane^47^, all within the range of 3–20 nm. Not only they include light and heavy components, but they also represent both hydrocarbon and non-hydrocarbon families of commonly investigated fluids, especially CO_2._ These 18 points are regarded as the test points of our work, constituting 20% of the whole 94 evaluated data points.

Two critical shift models presented by Zhang et al.^23^ (Eq. 49) and Yang et al.^31^ (Eq. 50) are employed herein for comparison. Both models incorporate the effect of adsorption thickness and are developed for the Lennard–Jones fluids.

49 $$\frac{{T}_{c}-{T}_{cp}}{{T}_{c}}=\frac{{P}_{c}-{P}_{cp}}{{P}_{c}}=0.7197\left(\frac{{\sigma }_{LJ}}{{r}_{p}-{\delta }_{p}}\right)-0.0758{\left(\frac{{\sigma }_{LJ}}{{r}_{p}-{\delta }_{p}}\right)}^{2}$$

50 $$\frac{{T}_{c}-{T}_{cp}}{{T}_{c}}=\frac{{P}_{c}-{P}_{cp}}{{P}_{c}}=3.374\times {\left(\frac{{r}_{p}-{\delta }_{p}}{{\sigma }_{LJ}}\right)}^{-1.637}$$

**Figure 11:**
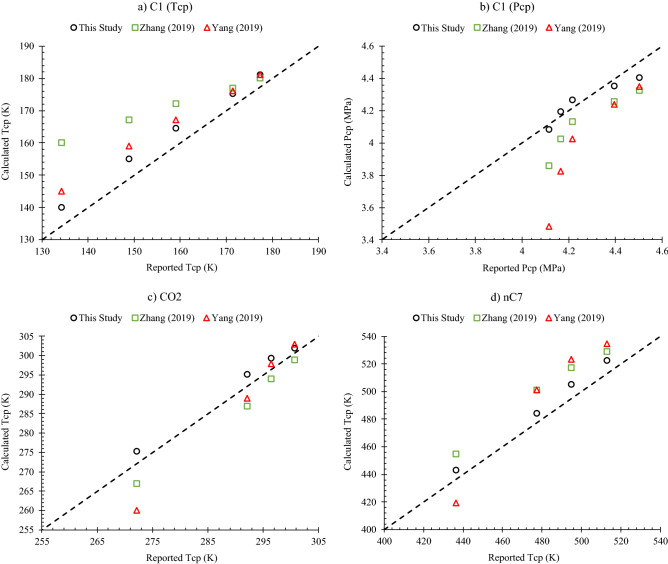
Cross plots of critical shift estimations from this study along with two other models in the literature 23,31 for (**a**, **b**) methane 46, (**c**) carbon dioxide, and (**d**) normal heptane 47.

As plotted in Fig. 11, the model developed in this study with its pore-size-dependent energy parameter has performed much better than other models in predicting the effect of confinement on fluids critical properties. This was more outstanding for critical pressure in particular, because no training data of P_cp_ has been included during model development in the previous section. The average deviation of our model was just below 2%, whereas the models of Zhang 2019 and Yang 2019 had the average errors of 4.4% and 4.5%, respectively.

### Phase diagrams and discussion on capillary condensation occurrence

Pressure–Volume isotherm diagrams of the van der Waals and Peng-Robinson (PR)^48^ EOSs were evaluated in the literature where they were associated with the Lennard–Jones potential function (LJ-vdW and LJ-PR). In spite of the LJ + vdW^6^, as it is controversially reported by Islam et al.^33^, the P–V axial isotherms of LJ + PR do not show capillary condensation at a same reduced temperature (${T}_{r}=T/{T}_{c}$) and the confined fluid’s phase appears to be constantly supercritical. To scrutinize this issue for the Kihara potential function, we will first draw a comparison between the PV isotherm diagrams of Kihara + vdW and Kihara + PR models at different ${T}_{r}$ values. Then, the effect of potential type and employing pore-size-dependent ${\varepsilon }_{k}$ on maxwell construction will be investigated.

#### P–V isotherms diagrams of Peng-Robinson vs. van der Waals

In order to incorporate the critical shift phenomena into EOS calculations, we replace the ${T}_{c}$ and ${P}_{c}$ parameters in the attractive ($a$) and repulsive ($b$) terms of EOSs with the shifted version derived from this study (${T}_{cp}, {P}_{cp}$). In this section, the ${\varepsilon }_{kp}$ concept is employed and its value is computed by Eq. (46), using the $\alpha$ and $\beta$ presented in Table 7.

**Figure 12:**
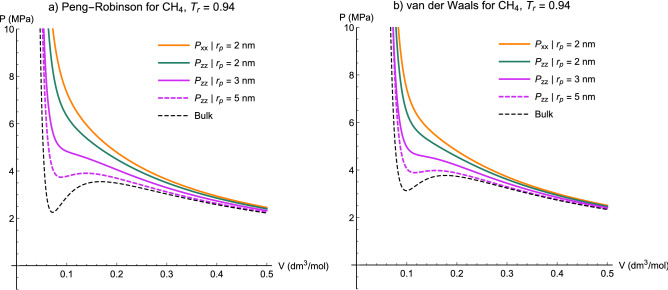
Volume dependence of the axial and transverse pressure tensor components of methane at ${T}_{r}=0.94$ for different ${r}_{p}$ values obtained from (**a**) Peng-Robinson and (**b**) van der Waals equations of state associated with the Kihara potential function.

We slightly reduce the temperature from the critical point region for methane, as an example. Figure 12 shows how the confinement influences the isotherm curves and the prediction of fluid’s state. Both vdW and PR equations of state perfectly expose the presence of Maxwell construction for the bulk fluid (black dashed line), and also the tendency of its confined case at ${r}_{p}=5 nm$ (purple dashed line) to act the same. However, the critical temperature at ${r}_{p}=2 nm$ is less than that of the current system, therefore the fluid must be homogenous but supercritical. This is well supported by the solid green and orange lines for the axial and transverse pressures, respectively, where ${P}_{zz}<{P}_{xx}$. It is well worthy to mention that the vdW EOS generates a smaller two-phase region, or in fact, less ${v}_{g}$. Consequently, isotherm lines of this EOS are more inclined towards showing capillary condensation sooner than their correspondents of PR.

**Figure 13:**
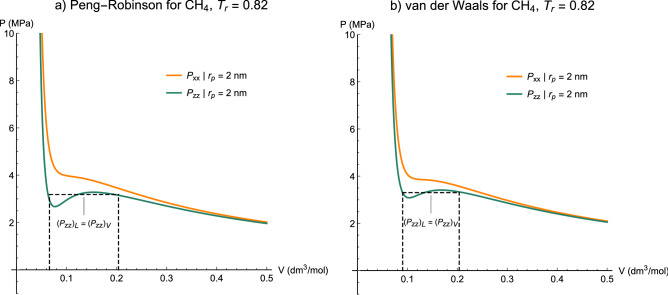
Capillary condensation of axial pressure for methane at ${T}_{r}=0.82$ obtained from (**a**) Peng-Robinson and (**b**) van der Waals equations of state associated with the Kihara potential function. Dashed line evidence the Maxwell construction for two-phase equilibrium.

**Figure 14:**
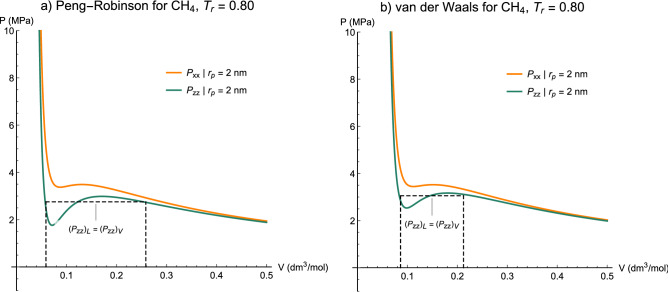
Capillary condensation of axial pressure and the tendency of the transverse pressure to construct the two-phase region for methane at ${T}_{r}=0.80$ obtained from (**a**) Peng-Robinson and (**b**) van der Waals equations of state associated with the Kihara potential function. Dashed line evidence the Maxwell construction for two-phase equilibrium.

Decreasing the temperature and maintaining the transverse $A$ constant, we observe a loop appearing for the axial pressure ${P}_{zz}$ when $T$ is less than ${T}_{cp}$ at 2 nm (Fig. 13). If we take the temperature even lower, the transverse pressure ${P}_{xx}$ also commences to presents a loop as well as the axial part (Fig. 14), which indicates both Kihara + vdW and Kihara + PR models reveal capillary condensation in the axial part at the same reduced temperature. A Video file is provided as supplementary material (which completely embeds the motion of P–V isotherms of Kihara-PR for methane through Figs. 12, 13, 14), so that the occurrence of capillary condensation of both bulk and confined fluids will be readily understandable.

#### The effect of $U({r}_{12})$ and ${\varepsilon }_{kp}$

The effect of potential function type and its variable energy parameter on Maxwell construction in P–V diagram is thoroughly explored in Supporting Information 3. Overall, it is interesting that the isotherm curves of Peng-Robinson equation of state when coupled with the Lennard–Jones potential function, perfectly shows capillary condensation for both axial and transverse components of the pressure tensor when the temperature is decreased. This is properly in contrast to what has been reported and explained in the literature by Islam et al.^33^. Moreover, at each ${T}_{r}$, the Kihara-PR model with ${\varepsilon }_{kb}$ results in widest two-phase regions compared to those of the Lennard–Jones since the use of constant energy parameter leads to greater ${T}_{cp}$ predictions. By applying ${\varepsilon }_{kp}$, the diagrams seem more rational and reliable in terms of the involved ${T}_{cp}$, and of course, they do not readily fail in the demonstration of capillary condensation unless at relatively low temperatures.

### Model validation (Application in mixtures equilibrium)

After we gained a better understanding of intermolecular forces and fluids’ molecular behavior by the proposed model, the best practice to predict and interpret the properties of mixtures would be applying the thermodynamic methods to phase equilibrium problems 20. In this section the validation of the proposed theory will be examined. Zhang et al.^34^ have modified the Peng-Robinson equation of state through involving the L-J critical shift model^6^, and employed their model to predict the equilibrium properties of a confined ternary system comprising i-C4, n-C4, and n-C_8_ in micro/nano-scale channels of $10 \mu m$ and 100 $nm$ ^49^.

Similarly, we implement the flash calculations on this system using the analytical model we have derived for the critical shift along with the proposed approach of pore-size-dependent energy parameter.

The details of the vapor–liquid equilibrium (VLE) calculations we have utilized in our approach are presented in Supporting Information 3. The results are listed and compared to the experimental reports^49^ and the results of Zhang et al., in Table 8^34^. As the table presents, our calculations are more agreeable with the true values, and our model have reduced the error of Lennard–Jones model by more than 6%.

**Table 8:** Experimental VLE properties of a ternary system *49* compared to the reported calculations of L-J model *34* and this model proposed in this study for 1-Constant Pressure Case and 2- Constant Temperature Case in 10 $\mu m$ and 100 nm channels.

Parameters	Before flash^49^			After flash^49^			After flash (L–J)^34^				After flash (Kihara) – This study			
1- Constant pressure case														
Temperature ($^\circ{\rm C} )$	24.9			71.9										
Pressure (Pa)	85,260									AARD (%)				AARD (%)
Liquid (iC4, nC4, C8) mol %	15.47	4.53	80	4.88	1.87	93.25	5.09	1.68	93.23	4.83	4.8333	1.8484	93.320	0.728
Vapor (iC4, nC4, C8) mol %	0	0	0	64.35	16.82	18.83	62.84	17.56	19.6	3.61	64.072	16.783	19.146	0.778
Liquid fraction (mol %)	100			82.2			82.03			0.21	82.044			0.190
Vapor fraction (mol %)	0			17.8			17.97			0.96	17.956			0.875
IFT ($mJ/{m}^{2}$)				16.24			15.68			3.45	14.890			8.313
${P}_{cap}$ micro-channel (kPa)				3.38			1.92			43.20	2.579			23.70
${P}_{cap}$ nano-channel (kPa)				286.91			185.63			35.30	231.12			19.44
Average %										13.08				7.72
2- Constant temperature case														
Temperature ($^\circ{\rm C} )$	71.9													
Pressure (Pa)	839,925			426,300										
Liquid (iC4, nC4, C8) mol %	61.89	18.11	20	28.59	11.15	60.26	18.98	6.44	74.58	33.21	28.598	11.149	60.254	0.017
Vapor (iC4, nC4, C8) mol %	0	0	0	75.82	21.01	3.16	76.66	19.35	3.99	11.76	75.798	21.018	3.1833	0.269
Liquid fraction (mol %)	100			29.5			30.38			2.98	29.467			0.113
Vapor fraction (mol %)	0			70.5			69.62			1.25	70.533			0.047
IFT ($mJ/{m}^{2}$)				13.33			12.89			3.30	12.217			8.51
${P}_{cap}$ micro-channel (kPa)				2.77			1.88			32.13	2.116			23.61
${P}_{cap}$ nano-channel (kPa)				235.54			182.49			22.52	183.13			22.25
Average %										15.31				7.81

## Summary and conclusion

Critical shift and phase transition of confined fluids have continually been a focal point of interest in a multitude of studies conducted with the help of molecular thermodynamics science. Yet, the lack of a better and more precise fundamental approach has always been noticed.

Overall, the following points can be concluded from the breakthroughs achieved in the fundamentals from this study:

- In spite of the Lennard–Jones (L–J) model, the energy integral should be calculated exclusively for each fluid when using the Kihara potential function, thereby leading to more accurate predictions. In this regard, each component has its specific integration constants (C_0_, C_1_, and C_2_), whereas the study for L–J proposes one unique set of constants for all fluids.
- The exact analytical solution to the second virial coefficient formula of Kihara fluids is presented for the very first time and the Kihara parameters are determined based on $B(T)$ data fitting with which the value of reduced Kihara parameter is obtained.
- The model must be able to render the bulk fluid’s critical properties when the pore radius approaches infinity, whereas it is not. The treatment of addressing this issue is called ‘**Adjustment’.** In the previous work of L-J, the adjustment was merely a correlation-based enhancement and lacked a thermodynamic basis. In our model, however, the values of Kihara parameters are determined in a way that the critical shift equation leads to the bulk critical properties of matters.
- In comparison to the experimental measurements, the exact solution of both models (Kihara and L-J) does not lead to acceptable predictions of ${T}_{cp}$. Hence, a novel idea is proposed and justified in which the energy parameter of Kihara fluids (${\varepsilon }_{k}$) varies with respect to pore size reduction (${\varepsilon }_{kp}$) and equated the bulk state $({\varepsilon }_{kb}$) at ${r}_{p}\to \infty$.

Finally, the proposed idea is supported by a collection of 94 critical shift data points and its performance is validated by evaluating the VLE properties of a ternary mixture, where our error was almost half of the L-J’s.

The followings are the advantages, disadvantages/limitations of this work along with suggestions for probable future works:

- *Advantages* The critical shift modeling is done exactly without any simplifying assumptions or approximations. This work benefits from a better adjustment method than the previous works of L-J. The notion of pore-size-dependent energy parameter is presented for the very first time that enables researchers to achieve the most precise predictions of confined fluid’s behavior providing enough experimental values are available. Employing the Kihara potential function gives more accurate results and better flexibility rather than the Lennard–Jones.
- *Disadvantages/Limitations* The pore critical temperature cannot be directly calculated since its formula is a little complex, and a root finding process should be followed. However, a precalculated type-curve was presented for convenience and ease of use. For similar works, sufficient number of experimental data points will be needed to calibrate the energy parameter of potential function.
- *Suggestions for future works* It is recommended to calibrate the energy parameter with pore critical pressure data, providing sufficient number of data points are accessible, to determine whether the results are far from those calibrated by pore critical temperature in this work.

## Supplementary Information

Supplementary Information 1.

Supplementary Information 2.

Supplementary Information 3.

Supplementary Information 4.

Supplementary Information 5.

Supplementary Video 1.

## Data Availability

The data of this study will be available upon request via contacting the corresponding author (m.khorsand@aut.ac.ir).

## List of symbols

${a}_{k}$: Kihara spherical core radius
${a}_{k}^{*}$: Reduced Kihara size parameter
${a}_{PR}$: Peng-Robinson constant
${a}_{vdW}$: Van der Waals constant
A: Transverse pore reduced area
B(T): Second virial coefficient
${b}_{PR}$: Peng-Robinson constant
${b}_{vdW}$: Van der Waals constant
C: Coefficient
E: Internal energy
$f(T)$: Free energy of ideal gas
F: Helmholtz free energy
H: Hermite function
I: Integral numerical solution
k: Boltzmann constant
L: Length
m: Coefficient
MW: Molecular weight
n: Coefficient
N: System particles number
$${N}_{A}$$: Avogadro constant
${p}_{ii}$: Component of pressure tensor
P: System pressure
$$\overrightarrow{P}$$: Pressure tensor
P_c_: Bulk critical pressure
r: Distance between molecular centers
$${r}_{p}$$: Pore size
S: Entropy
T: System temperature
$${T}^{*}$$: Reduced temperature
T_c_: Bulk critical temperature
T_cc_: Capillary critical temperature
T_cp_: Pore critical temperature
T_r_: Bulk reduced temperature
U: Internal energy
$$U({r}_{12})$$: Potential function
$$v$$: Molar volume
V: System volume

## Greek symbols

$$\alpha$$: Coefficient $$\beta : 1/kT$$
$$\Gamma$$: Gamma function
$$\gamma$$: Coefficient
$${\delta }_{p}$$: Adsorption thickness
$\varepsilon$: Kihara energy parameter ${\varepsilon }_{k}: \varepsilon /k$
$${\varepsilon }_{kb}$$: Bulk $${\varepsilon }_{k}$$
$${\varepsilon }_{kp}$$: $${\varepsilon }_{k}$$ In pores
${\epsilon }_{ii}$: Tensor of volume deformation
$$\lambda$$: Coefficient
$$\mu$$: Viscosity
$$\sigma$$: Size parameter
$$\omega$$: Acentric factor

## Subscripts

c: Critical
ii: Direction of a tensor’s components
$$k$$: Kihara
LJ: Lennard–Jones
p: Pore
PR: Peng-Robinson
vdW: Van der Waals
