# Supplementary material for: Critical temperature shift modeling of confined fluids using pore-size-dependent energy parameter of potential function
Source: Sci Rep. 2023 Mar 24;13:4842. doi: 10.1038/s41598-023-31998-7 (PMC10039086; doi:10.1038/s41598-023-31998-7)
Supplement: Supplementary file 1 — Supplementary Information 1. [file 41598_2023_31998_MOESM1_ESM.pdf]

Supporting Information 1

Critical Temperature Shift Modeling of Confined Fluids Using Pore-Size-Dependent Energy Parameter of Potential Function

by

Mohammad Humand, Mohammad Reza Khorsand Movaghar*

Correspondence: m.khorsand@aut.ac.ir

Department of Petroleum Engineering

Amirkabir University of Technology

Tehran, Iran

Analytical expression of C_0 as a function of $B(T)$

From the bulk perspective and based on the partition function theory we have [1],

$$B(T)_{bulk} = 2\pi N_A \int_0^\infty \left(1 - e^{-\beta U(r_{12})}\right) r^2 dr. \qquad \text{S1. 1}$$

With the help of Mathematica software version 12.1 [2], the exact analytical solution of S1. 1 is

$$2\pi(\sigma_k - 2a_k)^3 N_A \times \left(-\frac{\left(\frac{T}{\varepsilon_k}\right)^{\frac{3}{4}} \Gamma\left(-\frac{1}{4}\right) F_1\left(-\frac{1}{4}, \frac{1}{2}, \frac{\varepsilon_k}{T}\right) + 2\left(\frac{T}{\varepsilon_k}\right)^{\frac{1}{4}} \Gamma\left(\frac{1}{4}\right) F_1\left(\frac{1}{4}, \frac{3}{2}, \frac{\varepsilon_k}{T}\right)}{6\sqrt{2}\frac{T}{\varepsilon_k}} \right.$$
$$+ \frac{2 \times 2^{\frac{1}{3}} \times a_k \left(\frac{\varepsilon_k}{T}\right)^{\frac{2}{3}} \left[\sqrt{\frac{T}{\varepsilon_k}} \Gamma\left(\frac{5}{6}\right) F_1\left(-\frac{1}{6}, \frac{1}{2}, \frac{\varepsilon_k}{T}\right) - \Gamma\left(\frac{4}{3}\right) F_1\left(\frac{1}{3}, \frac{3}{2}, \frac{\varepsilon_k}{T}\right)\right]}{(\sigma_k - 2a_k)} \qquad \text{S1. 2}$$
$$\left. + \frac{2 \times 2^{\frac{1}{6}} \times a_k^2 \left(\frac{\varepsilon_k}{T}\right)^{\frac{7}{12}} \left[6\sqrt{\frac{T}{\varepsilon_k}} \Gamma\left(\frac{11}{12}\right) F_1\left(-\frac{1}{12}, \frac{1}{2}, \frac{\varepsilon_k}{T}\right) - \Gamma\left(\frac{5}{12}\right) F_1\left(\frac{5}{12}, \frac{3}{2}, \frac{\varepsilon_k}{T}\right)\right]}{(\sigma_k - 2a_k)^2} \right)$$

where and Γ is the Gamma function, and F_1 is the Hypergeometric1F1 function.

After using conversions S1. 3 to S1. 5, we are given by S1. 6.

$$H_n(z) = 2^n U\left(-\frac{1}{2}n, \frac{1}{2}, z^2\right) \qquad \text{S1. 3}$$

$$U(a, b, z) = \sqrt{\pi}\csc(\pi b)\left[\frac{F_1(a, b, z)}{\Gamma(a - b + 1)} + 2z^{1-b}\frac{F_1(a - b + 1, 2 - b, z)}{\Gamma(a)}\right] \qquad \text{S1. 4}$$

$$\Gamma(x)\Gamma(-x) = \frac{-\pi}{x\sin(\pi x)} \quad , \Gamma(x)\Gamma(1 - x) = \frac{\pi}{\sin(\pi x)} \quad , \Gamma(1 + x) = x\Gamma(x) \qquad \text{S1. 5}$$

$$B(a_k^*,T^*)_{bulk} = 2\pi N_A \sigma_k^3 (1-a_k^*)^3 \times \left(\frac{-\left(\frac{1}{T^*}\right)^{\frac{1}{4}}}{12\sqrt{\pi}} \left(\Gamma\left[-\frac{1}{4}\right]\Gamma\left[\frac{1}{4}\right]\right) H_{\frac{1}{2}}\left[-\sqrt{\frac{1}{T^*}}\right] + \frac{3\times a_k^* \left(\frac{1}{T^*}\right)^{\frac{1}{6}}}{\sqrt{\pi}(1-a_k^*)} \left(\Gamma\left[\frac{5}{6}\right]\Gamma\left[\frac{4}{3}\right]\right) H_{\frac{1}{3}}\left[-\sqrt{\frac{1}{T^*}}\right] + \frac{{a_k^*}^2 \left(\frac{1}{T^*}\right)^{\frac{1}{12}}}{\sqrt{\pi}(1-a_k^*)^2} \left(\Gamma\left[\frac{11}{12}\right]\Gamma\left[\frac{5}{12}\right]\right) H_{\frac{1}{6}}\left[-\sqrt{\frac{1}{T^*}}\right] \right) \quad S1.6$$

in which H_n is the Hermite polynomial, U is the Hypergeometric function, $a_k^* = 2a_k/\sigma_k$ is the reduced Kihara parameter, and $T^* = T/\varepsilon_k$ is the reduced temperature.

In the confined perspective, we split the integral region in S1. 1 into two parts. Ignoring the region $0 \le r_{12} < 2a_k$ since it has no physical meaning according to the Kihara potential's definition, we have,

$$B(T)_{conf} = 2\pi N_A \left(\int_{2a_k}^{\sigma_k} \left(1-e^{-\beta U(r_{12})}\right) r^2 dr + \int_{\sigma_k}^{\infty} \left(1-e^{-\beta U(r_{12})}\right) r^2 dr \right) \quad S1.7$$

The first part is

$$\int_{2a_k}^{\sigma_k} \left(1-e^{-\beta U(r_{12})}\right) r^2 dr = \frac{N_A}{2} \times \frac{4\pi}{3} \sigma_k^3 \left(1-{a_k^*}^3\right) \quad S1.8$$

and for the second we derive,

$$\int_{\sigma_k}^{\infty} \left(1-e^{-\beta U(r_{12})}\right) r^2 dr = \frac{N_A}{2} \times \sigma_k^3 \times C_o(a_k^*,T^*). \quad S1.9$$

After equating the two perspectives, the analytical equation for C_0 is determined by,

$$C_0(a_k^*, T^*) = \frac{2B(T^*)_{bulk}}{\sigma_k^3 \times N_A} - 2b^* \qquad S1.10$$

where $b^* = (2\pi/3)\left(1 - {a_k^*}^3\right)$.

Second Virial Coefficient Curve Fitting

The bulk analytical equation of the second virial coefficient (B(T)) based on the Kihara potential function is as follows,

$$\begin{aligned} B(a_k^*, T^*)_{bulk} &= 2\pi N_A \sigma_k^3 (1 - a_k^*)^3 \\ &\times \left(\frac{-\left(\frac{1}{T^*}\right)^{\frac{1}{4}}}{12\sqrt{\pi}} \left(\Gamma\left[-\frac{1}{4}\right]\Gamma\left[\frac{1}{4}\right]\right) H_{\frac{1}{2}}\left[-\sqrt{\frac{1}{T^*}}\right] \right. \\ &+ \frac{3 \times a_k^* \left(\frac{1}{T^*}\right)^{\frac{1}{6}}}{\sqrt{\pi}(1 - a_k^*)} \left(\Gamma\left[\frac{5}{6}\right]\Gamma\left[\frac{4}{3}\right]\right) H_{\frac{1}{3}}\left[-\sqrt{\frac{1}{T^*}}\right] \\ &+ \left. \frac{{a_k^*}^2 \left(\frac{1}{T^*}\right)^{\frac{1}{12}}}{\sqrt{\pi}(1 - a_k^*)^2} \left(\Gamma\left[\frac{11}{12}\right]\Gamma\left[\frac{5}{12}\right]\right) H_{\frac{1}{6}}\left[-\sqrt{\frac{1}{T^*}}\right] \right) \end{aligned} \qquad S2.11$$

where $a_k^* = 2a_k/\sigma_k$ is the reduced Kihara parameter, T^* is the reduced temperature, Γ is the Gamma function, H_n is the Hermite polynomial, and N_A is the Avogadro's number.

The following figures show the cross-plots of the second virial coefficient data fitting for the Kihara parameters based on the DIPPR database.

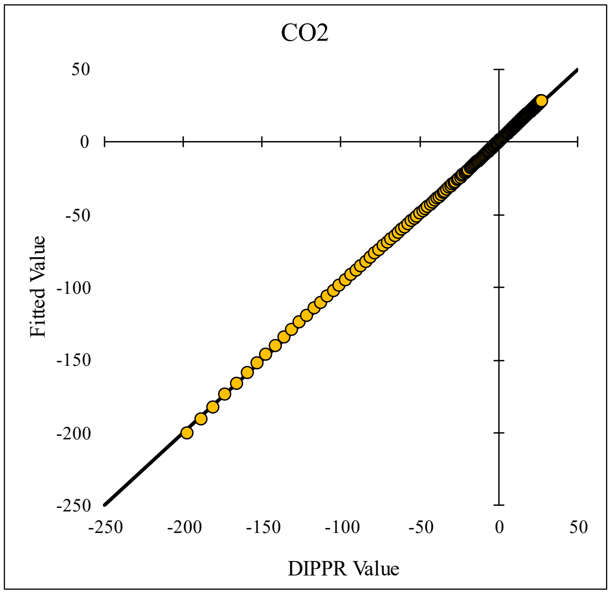

Fig.S1.1. Diagram of fitted data vs. DIPPR data of the second virial coefficient for CO_2. The solid line represents the $y = x$ line.

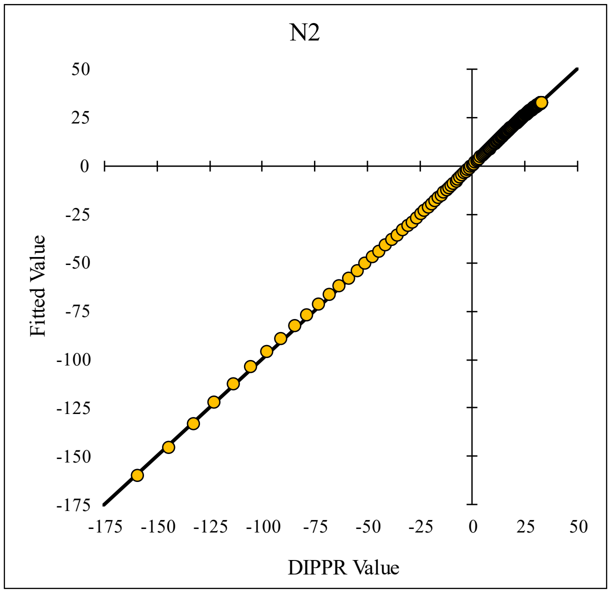

Fig.S1.2. Diagram of fitted data vs. DIPPR data of the second virial coefficient for N_2. The solid line represents the $y = x$ line.

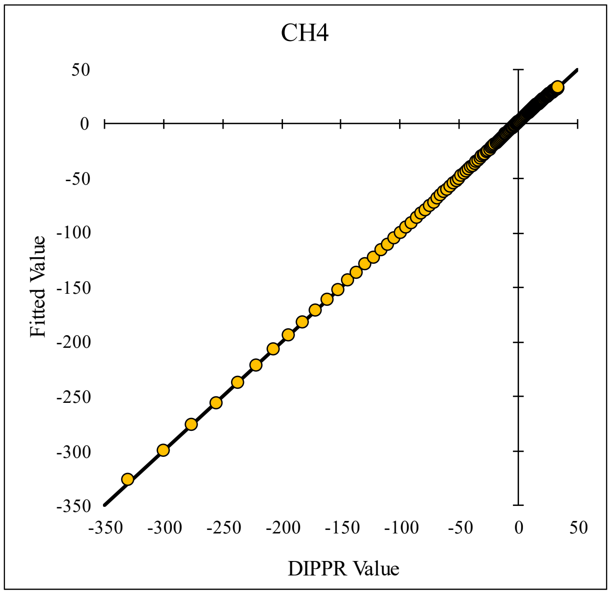

Fig.S1.3. Diagram of fitted data vs. DIPPR data of the second virial coefficient for CH_4. The solid line represents the $y = x$ line.

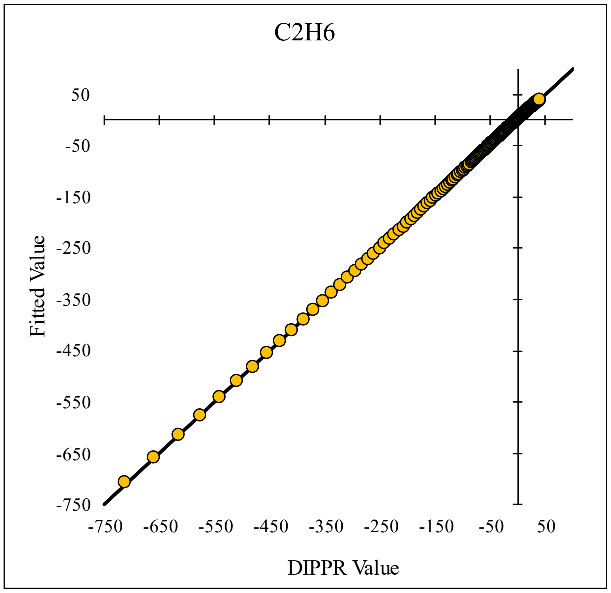

Fig.S1.4. Diagram of fitted data vs. DIPPR data of the second virial coefficient for C_2H_6. The solid line represents the $y = x$ line.

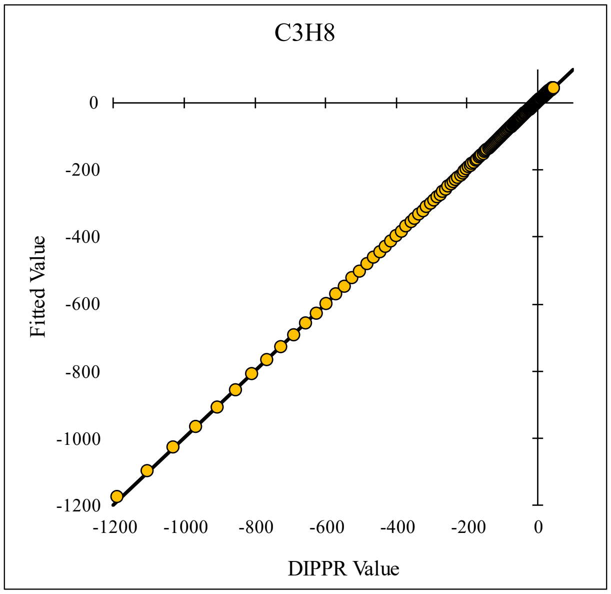

Fig.S1.5. Diagram of fitted data vs. DIPPR data of the second virial coefficient for C_3H_8. The solid line represents the $y = x$ line.

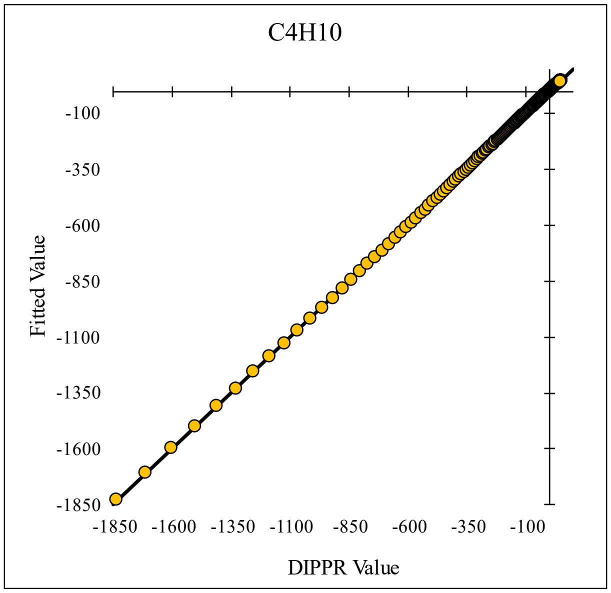

Fig.S1.6. Diagram of fitted data vs. DIPPR data of the second virial coefficient for C_4H_{10}. The solid line represents the $y = x$ line.

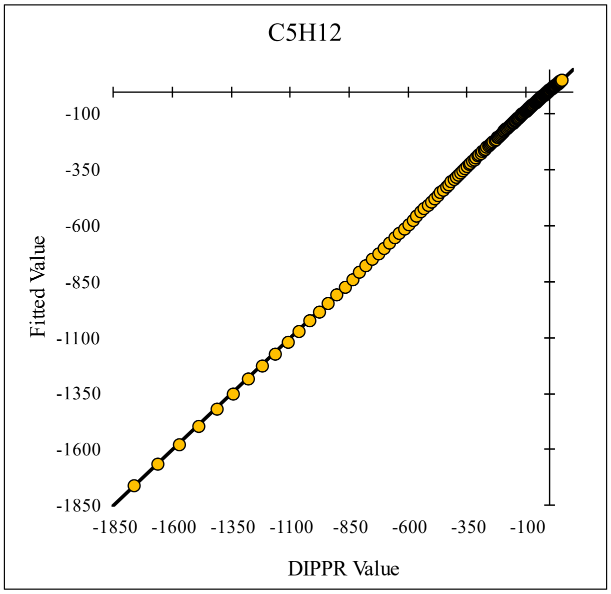

Fig.S1.7. Diagram of fitted data vs. DIPPR data of the second virial coefficient for C_5H_{12}. The solid line represents the $y = x$ line.

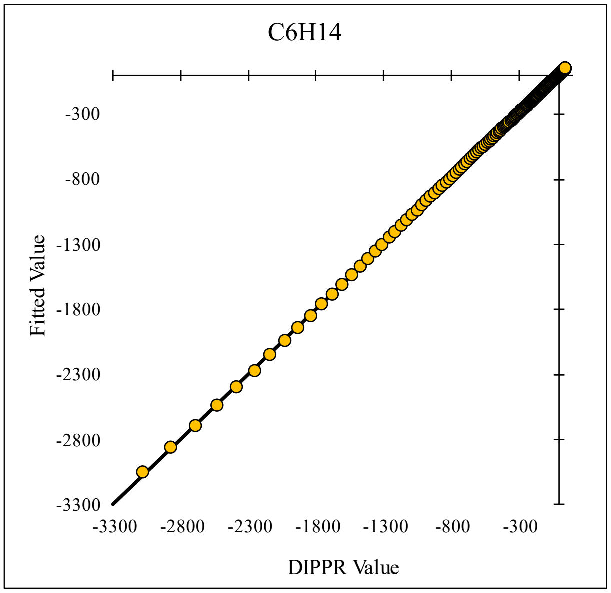

Fig.S1.8. Diagram of fitted data vs. DIPPR data of the second virial coefficient for C_6H_{14}. The solid line represents the $y = x$ line.

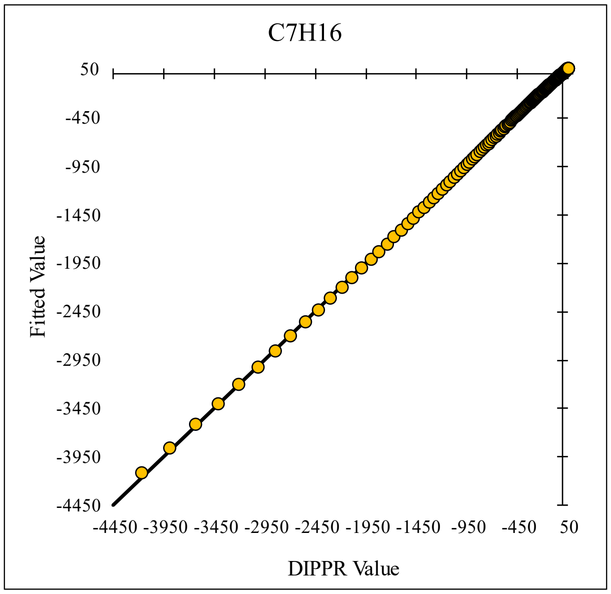

Fig.S1.9. Diagram of fitted data vs. DIPPR data of the second virial coefficient for C_7H_{16}. The solid line represents the $y = x$ line.

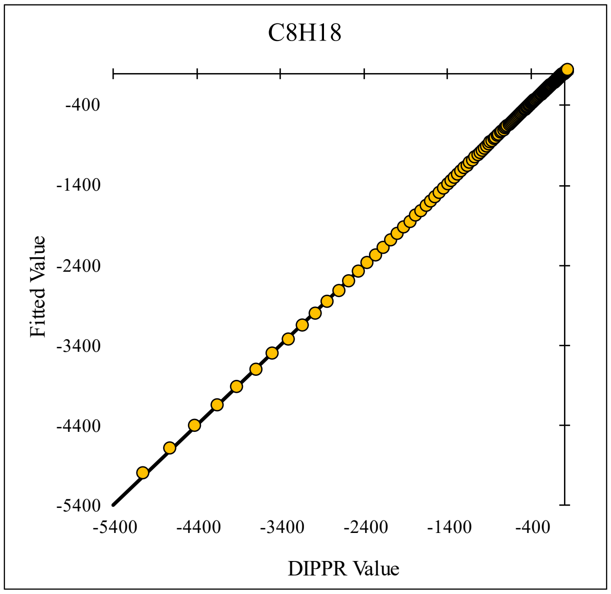

Fig.S1.10. Diagram of fitted data vs. DIPPR data of the second virial coefficient for C_8H_{18}. The solid line represents the $y = x$ line.

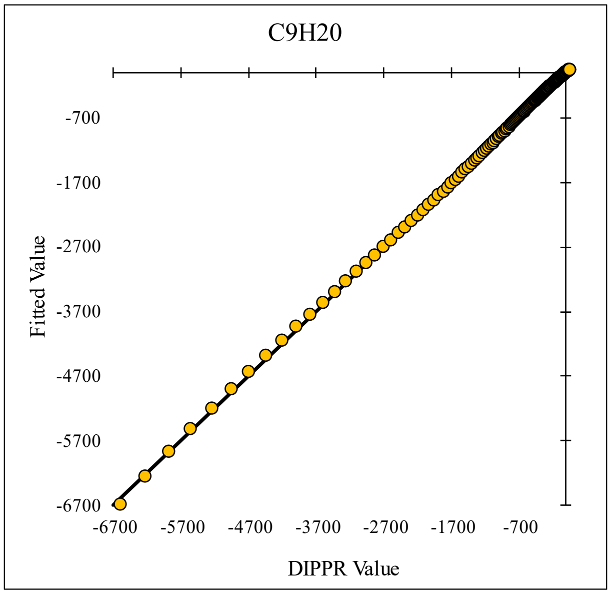

Fig.S1.11. Diagram of fitted data vs. DIPPR data of the second virial coefficient for C_9H_{20}. The solid line represents the $y = x$ line.

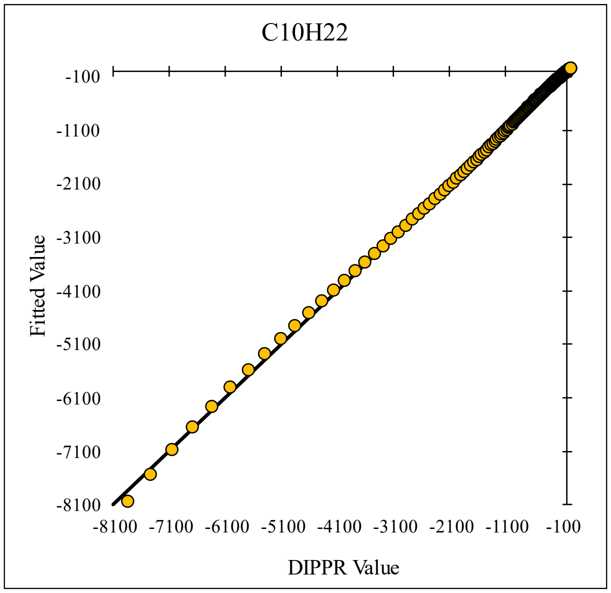

Fig.S1.12. Diagram of fitted data vs. DIPPR data of the second virial coefficient for $C_{10}H_{22}$. The solid line represents the $y = x$ line.

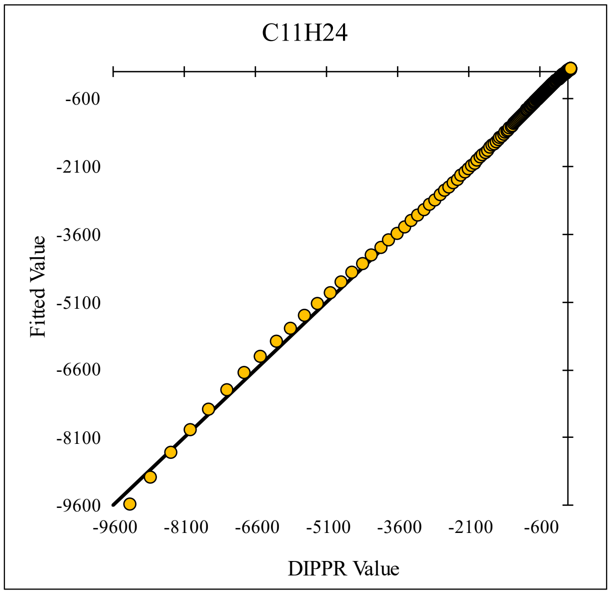

Fig.S1.13. Diagram of fitted data vs. DIPPR data of the second virial coefficient for $C_{11}H_{24}$. The solid line represents the $y = x$ line.

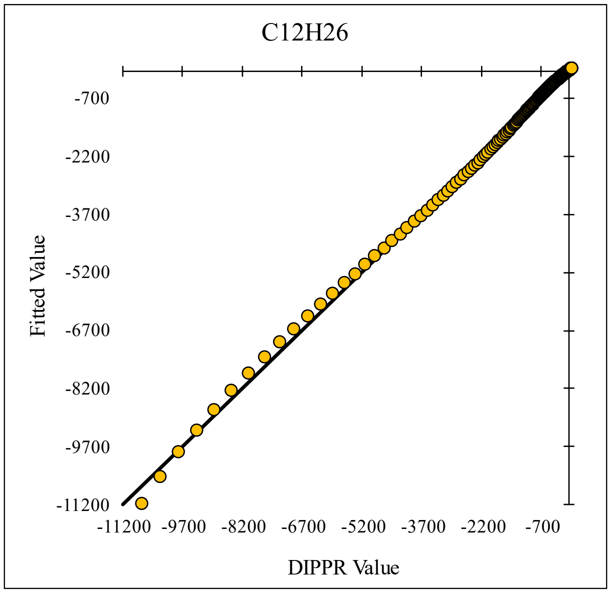

Fig.S1.14. Diagram of fitted data vs. DIPPR data of the second virial coefficient for $C_{12}H_{26}$. The solid line represents the $y = x$ line.

References

1 Hirschfelder, J. O., Curtiss, C. F., Bird, R. B. & Mayer, M. G. *Molecular theory of gases and liquids*. Vol. 165 (Wiley New York, 1964).

2 Wolfram Mathematica v. 12.1 (Wolfram Research, 2020) https://www.wolfram.com/mathematica.
