# Supplementary material for: Critical temperature shift modeling of confined fluids using pore-size-dependent energy parameter of potential function
Source: Sci Rep. 2023 Mar 24;13:4842. doi: 10.1038/s41598-023-31998-7 (PMC10039086; doi:10.1038/s41598-023-31998-7)
Supplement: Supplementary file 2 — Supplementary Information 2. [file 41598_2023_31998_MOESM2_ESM.pdf]

Supporting Information 2

Critical Temperature Shift Modeling of Confined Fluids Using Pore-Size-Dependent Energy Parameter of Potential Function

by

Mohammad Humand, Mohammad Reza Khorsand Movaghar*

Correspondence: m.khorsand@aut.ac.ir

Department of Petroleum Engineering

Amirkabir University of Technology

Tehran, Iran

Requirements of the equation of state (EOS derivatives)

The model derived from this study is

$$P_{zz}^* = \frac{T^*}{v^* - b^*} - \frac{T^*}{{v^*}^2}\left[-\frac{C_0(a_k^*, T^*)}{2} - \frac{1}{2}\left(\frac{C_1(a_k^*, T^*)}{\sqrt{A}} + \frac{C_2(a_k^*, T^*)}{A}\right)\right], \quad S2.1$$

where $P^* = P\sigma_k^3/\varepsilon$; $T^* = kT/\varepsilon$; $v^* = (V/N)\sigma_k^{-3}$; $b^* = (2\pi/3)\left(1 - {a_k^*}^3\right)$; and $a_k^* = 2a_k/\sigma_k$. For the vdW type EOSs, the following conditions are required [1]:

$$\left(\frac{\partial P_{zz}^*}{\partial v^*}\right)_{T^*=T_c^*} = \left(\frac{\partial^2 P_{zz}^*}{\partial {v^*}^2}\right)_{T^*=T_c^*} = 0 \quad S2.2$$

Applying S2. 2 on S2. 1 and considering the bulk state via $A \to \infty$, we have,

$$\left(\frac{\partial p_{zz}^*}{\partial v^*}\right)_{T_c^*} = -\frac{T_C^*}{(v_c^* - b^*)^2} - \frac{T_c^* \times C_0(a_k^*, T^*)}{{v_c^*}^3} = 0 \quad S2.3$$

$$-\frac{C_0(a_k^*, T^*)}{{v_c^*}^3} = \frac{1}{(v_c^* - b^*)^2} \quad S2.4$$

and,

$$\left(\frac{\partial^2 p_{zz}^*}{\partial {v^*}^2}\right)_{T_c^*} = \frac{2T_C^*}{(v_c^* - b^*)^3} + \frac{3T_c^* \times C_0(a_k^*, T^*)}{{v_c^*}^4} = 0 \quad S2.5$$

$$\frac{3 \times C_0(a_k^*, T^*)}{{v_c^*}^4} = -\frac{2}{(v_c^* - b^*)^3} \quad S2.6$$

Dividing two sides of S2. 6 by S2. 4 (or $\frac{\text{S2.6}}{\text{S2.4}}$),

$$-\frac{3}{v_c^*} = -\frac{2}{v_c^* - b^*} \quad S2.7$$

$$2v_c^* = 3v_c^* - 3b^* \quad S2.8$$

$$b^* = \frac{v_c^*}{3} \quad S2.9$$

Substituting b^* into S2. 4,

$$\frac{C_0(a_k^*, T^*)}{{v_c^*}^3} = \frac{-1}{\left(v_c^* - \frac{v_c^*}{3}\right)^2} = \frac{-9}{4{v_c^*}^2} \quad S2.10$$

$$C_0(T_c^*, a_k^*) = \frac{-9}{4} v_c^* = \frac{-27}{4} b^* \qquad S2.\,11$$

Substituting S2. 9 and S2. 11 into S2. 1,

$$p_c^* = \frac{T_c^*}{2b^*} + \frac{1}{9b^{*2}} \left(T_c^* \times \frac{\frac{-27}{4} b^*}{2} \right) = \frac{T_c^*}{2b^*} - \frac{1}{9b^{*2}} \left(T_c^* \times \frac{27b^*}{8} \right) \qquad S2.\,12$$

$$p_c^* = \frac{T_c^*}{2b^*} - \frac{3T_c^*}{8b^*} = \frac{T_c^*}{8b^*} \qquad S2.\,13$$

$$b^* = \frac{T_c^*}{8p_c^*} \qquad S2.\,14$$

$$b^* = \frac{\frac{T_c}{\varepsilon/k}}{8\, P_c \frac{\sigma_k^3}{\varepsilon}} \qquad S2.\,15$$

$$b^* = \frac{kT_c}{8P_c \sigma_k^3} \qquad S2.\,16$$

Finally, substituting S2. 16 in S2. 11,

$$C_0(a_k^*, T^*) = -\frac{27}{32} \frac{T_c^*}{p_c^*} \qquad S2.\,17$$

$$\frac{2B(a_k^*, T_c^*)_{bulk}}{\sigma_k^3 \times N_A} = -\frac{19}{32} \frac{kT_c}{P_c \sigma_k^3} \qquad S2.\,18$$

$$\frac{2B(a_k^*, T_c^*)_{bulk}}{N_A} = -\frac{19}{32} \times \frac{kT_c}{P_C} \qquad S2.\,19$$

in which the $B(a_k^*, T_c^*)_{bulk}$ is the bulk analytical equation of the second virial coefficient, presented in Supporting Information 1.

Fitting Kihara parameters based on the second virial coefficient data

In this study, the second virial coefficient reports of DIPPR database are employed to determine the Kihara parameters based on Equation S2.20 in Supporting Information 1. Table 1 lists the Kihara parameters of the mentioned components from $B(T)$ least-square fitting for a wide range of temperatures, all with R^2 of 0.999 (See Supporting Information 1). Figure 1 reveals for normal alkanes that a_k^* is closely correlated with $\omega \times MW$

Table 1. Values of Kihara parameters fitted with the second virial coefficient data.

**Component**	$\boldsymbol{a_k}$ [Å]	$\boldsymbol{\sigma_k}$ [Å]	$\boldsymbol{a_k^* = 2a_k/\sigma_k}$	$\boldsymbol{\varepsilon_k}$ $[\boldsymbol{K}]$	Temperature Range (K)
N_2	0.2944	3.534	0.1666	132.02	100 – 1500
CO_2	0.8854	3.661	0.4837	501.32	152.10 – 1500
CH_4	0.2692	3.699	0.1456	193.42	110.83 – 1500
C_2H_6	0.4988	4.176	0.2389	355.92	152.71 – 1500
C_3H_8	0.8490	4.617	0.3678	526.78	184.91 – 1500
C_4H_{10}	1.2364	5.076	0.4872	709.13	212.58 – 1500
C_5H_{12}	1.5477	5.413	0.5718	883.96	234.80 – 1500
C_6H_{14}	1.7957	5.986	0.6000	958.30	253.70 – 1500
C_7H_{16}	2.2680	6.499	0.6980	1137.97	270.10 – 1500
C_8H_{18}	2.1613	6.473	0.6678	1228.42	284.38 – 1500
C_9H_{20}	2.6800	7.254	0.7389	1338.75	297.83 – 1500
$C_{10}H_{22}$	3.0817	7.930	0.7772	1422.20	309.23 – 1500
$C_{11}H_{24}$	3.5694	8.762	0.8147	1494.85	319 – 1500
$C_{12}H_{26}$	4.6250	10.55	0.8767	1552.49	329 – 1500

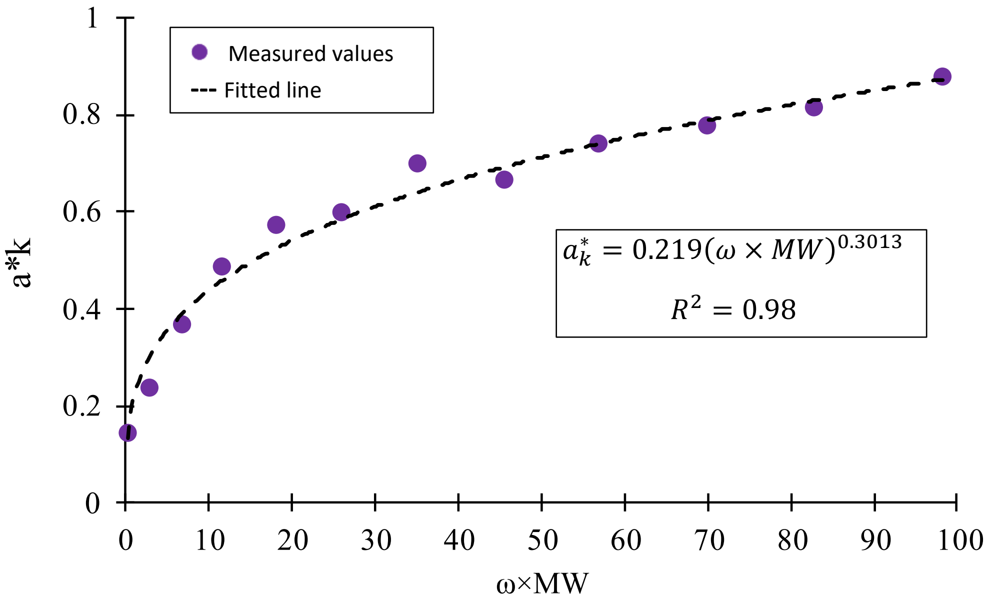

Figure 1. Values of a_k^* from the second virial coefficient data fitting vs. the multiplication of acentric factor and molecular weights of matters.

References

1 Danesh, A. *PVT and phase behaviour of petroleum reservoir fluids*. (Elsevier, 1998).
