# Supplementary material for: Critical temperature shift modeling of confined fluids using pore-size-dependent energy parameter of potential function
Source: Sci Rep. 2023 Mar 24;13:4842. doi: 10.1038/s41598-023-31998-7 (PMC10039086; doi:10.1038/s41598-023-31998-7)
Supplement: Supplementary file 3 — Supplementary Information 3. [file 41598_2023_31998_MOESM3_ESM.pdf]

Supporting Information 3

Critical Temperature Shift Modeling of Confined Fluids Using Pore-Size-Dependent Energy Parameter of Potential Function

by

Mohammad Humand, Mohammad Reza Khorsand Movaghar*

Correspondence: m.khorsand@aut.ac.ir

Department of Petroleum Engineering

Amirkabir University of Technology

Tehran, Iran

The effect of $U(r_{12})$ and ε_{kp} on Maxwell construction in P-V diagrams

Figure 1 demonstrates how the type of the potential function and the use of variable or constant ε_k can affect the shape of the isotherm lines in the pressure-volume diagrams. It is interesting that the isotherm curves of Peng-Robinson equation of state when coupled with the Lennard-Jones potential function, perfectly shows capillary condensation for both axial and transverse components of the pressure tensor when the temperature is decreased (Figure 1: a3, b3). This is properly in contrast to what has been reported and explained in the literature by Islam et. al. 1. For this model, however, the bulk or constant energy parameter (ε_k) were involved in the calculations to be better compared with the result of Islam et. al.

The differences among the cases are distinguishable when the temperature declines even more. At each T_r, the Kihara-PR model with ε_{kb} (Figure 1: a2, b2) results in widest two-phase regions compared to those of the Lennard-Jones since the use of constant energy parameter leads to greater T_{cp} predictions. Using ε_{kp}, the diagrams seem more rational and reliable in terms of the involved T_{cp}, and of course, they do not readily fail in the demonstration of capillary condensation unless at relatively low temperatures (Figure 1: a1, b1).

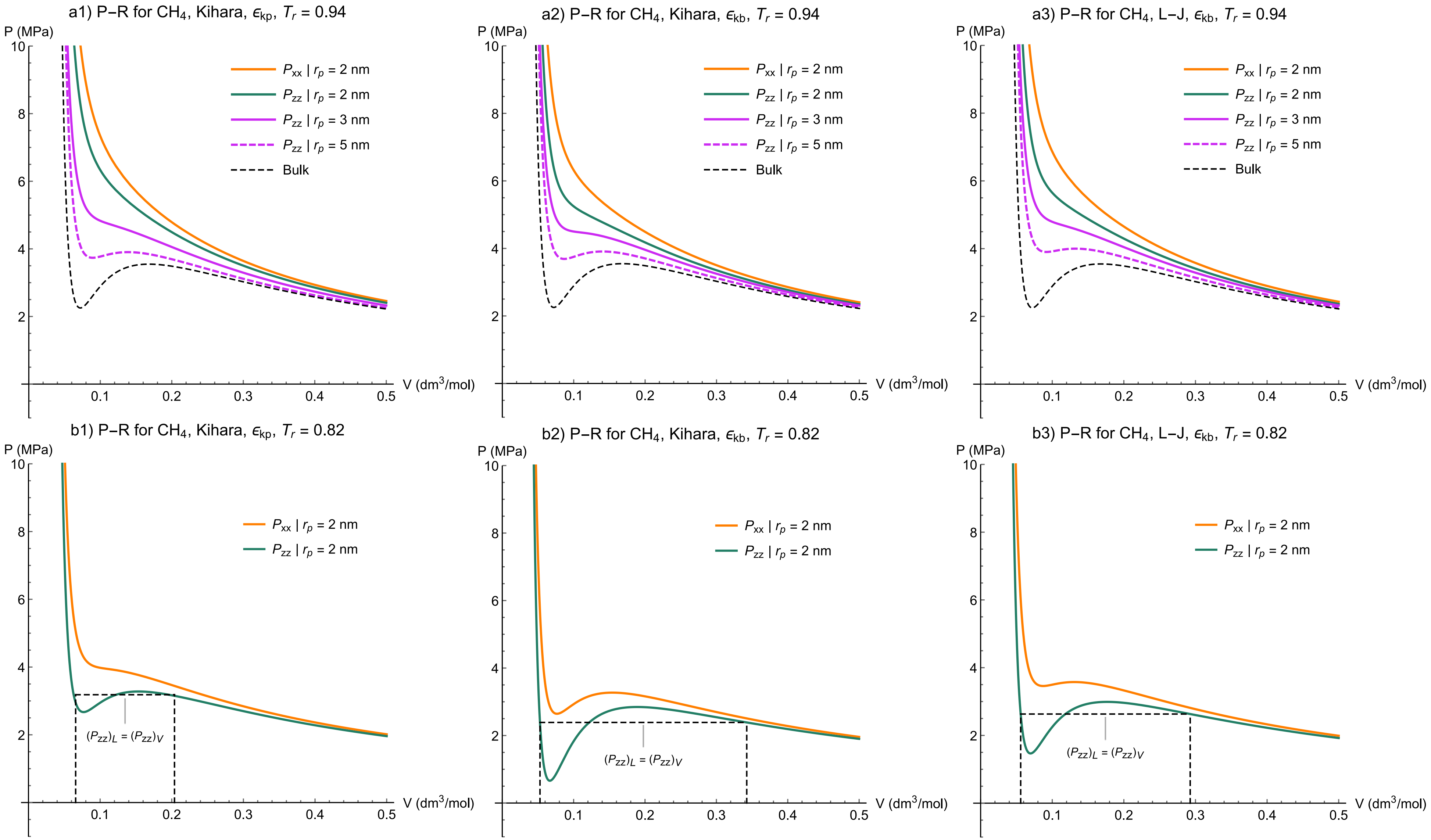

Figure 1. Comprehensive depiction of the effect of potential type and the employment of ε_{kp} on P-V isotherm diagrams with respect to temperature reduction. **a1, b1)** The Kihara-PR model employed with pore-dependent energy parameter (ε_{kp}) using Equation **Error! Reference source not found.** (original paper), which looks much more reasonable. **a2, b2)** The K ihara-PR model employed with constant energy parameter at the bulk state (ε_{kb}) derived from the adjustment stage for Kihara (Table 6 of the original paper). **a3, b3)** The LJ-PR model employed with ε_{kb} derived from the adjustment stage for L-J (Table 4 of the original paper) which is opposite to what has been reported for this model.

Details of two-phase flash calculations

The first step in the flash calculations is to guess an initial value for K values for which we have used the Wilson equation [2]:

$$K_i = \frac{y_i}{x_i} = \frac{P_{ci}}{P} \exp\left[5.37(1+\omega_i)\left(1-\frac{T_{ci}}{T}\right)\right] \quad (1)$$

where T_{ci} and P_{ci} are the bulk critical temperature and pressure of the component i, and ω is the acentric factor. Yet, in order to consider the critical shift effect, T_c and P_c must be replaced by T_{cp} and P_{cp}, respectively, calculated based on the approach presented in this study.

Mass transfer on one mole of mixture is as follows,

$$Z_i = x_i l + y_i(1-l) \quad (2)$$

in which Z_i is the mole fraction of component i in the original mixture, l is the mole fraction of liquid phase, and x_i and y_i are the liquid and vapor molar fractions of the component i. Combining 1 and 2, we have

$$x_i = \frac{Z_i}{1+(1-l)K_i} \quad (3)$$

$$y_i = \frac{K_i Z_i}{1+(1-l)K_i} \quad (4)$$

The summation of components mole fraction in each phase must be equal to 1, therefore 5 known as the Rachford-Rice equation [3] is achieved.

$$f(l) = \sum_{i}^{N_c} \frac{Z_i(1-K_i)}{K_i+(1-K_i)l} = 0 \quad (5)$$

The system is in equilibrium when the following is true for all components.

$$\hat{f}_i^l = \hat{f}_i^v, \ \ i = 1,2,\dots,N_c \quad (6)$$

Where $\hat{f}_i^l$ and $\hat{f}_i^v$ are the fugacity of liquid and vapor phases, respectively. Numerically, this is equivalent to,

$$\left|\frac{\hat{f}_i^l}{\hat{f}_i^v}\right| < \varepsilon' \qquad 7$$

Here ε' is a small number, usually in the range of 10^{-4} to 10^{-6}.

The capillary pressure is determined as 4,

$$P_{cap} = P_v - P_l = \frac{2\sigma}{r}\cos\theta \qquad 8$$

where P_{cap} is the capillary pressure, P_g and P_l are the vapor phase and liquid phase pressures, respectively, σ is the interfacial tension (IFT), r is the pore size, and θ is the wettability angle which is considered 30 degrees in this work. In this study, first P_l is calculated and P_v is determined through $P_v = P_l + P_{cap}$. Moreover, IFT is calculated as follows,

$$\sigma = \left[\sum_i \gamma_i (x_i \rho^l - y_i \rho^v)\right]^4 \qquad 9$$

in which γ_i is the Parachor value of component i, ρ^l and ρ^v are the vapor and liquid phases densities, respectively 4. shows the flash calculations flowchart employed in this work.

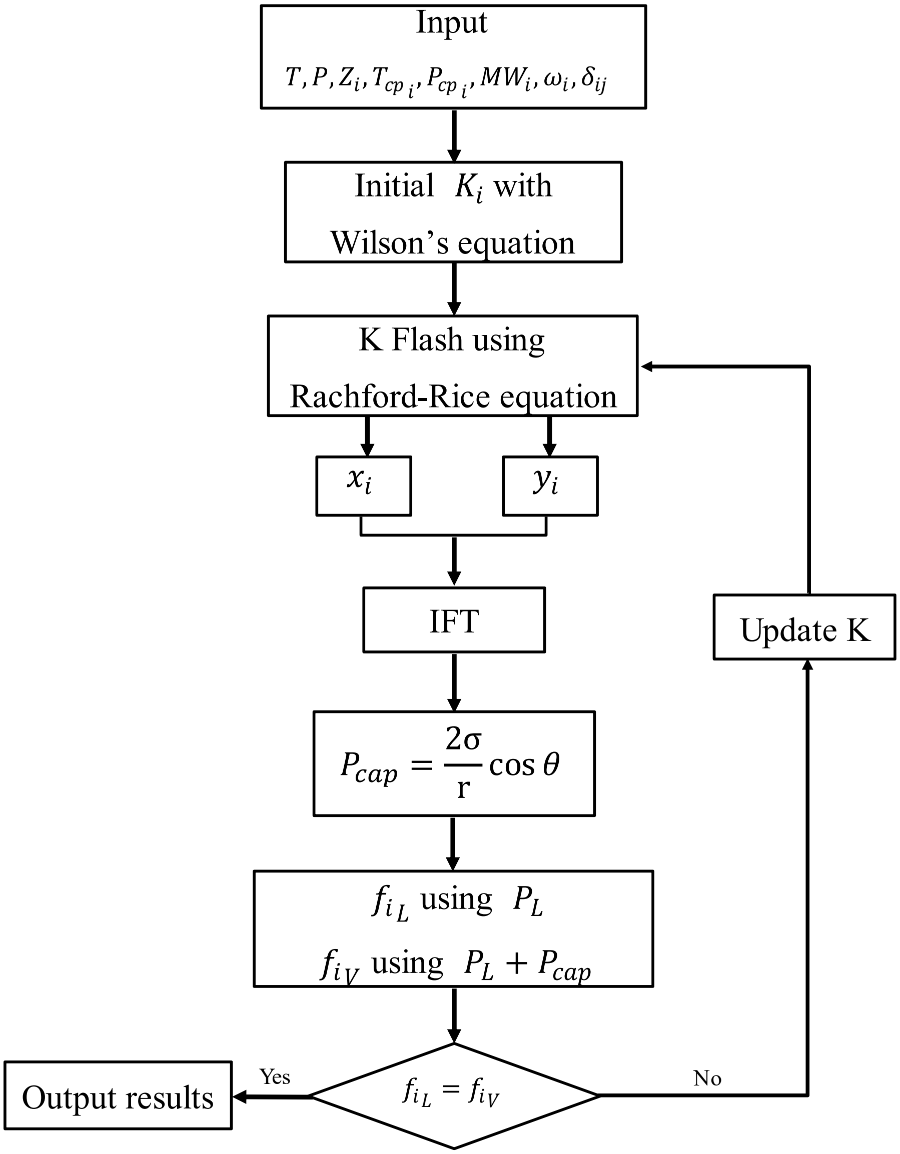

Figure 2 Flowchart of the flash calculation employed in this study.

References:

1 Islam, A. W., Patzek, T. W. & Sun, A. Y. Thermodynamics phase changes of nanopore fluids. *Journal of Natural Gas Science and Engineering* **25**, 134-139 (2015).

2 Wilson, G. M. in *65th National AIChE Meeting, Cleveland, OH.* 15.

3 Rachford Jr, H. & Rice, J. Procedure for use of electronic digital computers in calculating flash vaporization hydrocarbon equilibrium. *Journal of Petroleum Technology* **4**, 19-13 (1952).

4 Haider, B. A. *Impact of capillary pressure and critical properties shift due to confinement on hydrocarbon production from shale reservoirs*, Standford University, (2015).
