# Supplementary material for: Critical temperature shift modeling of confined fluids using pore-size-dependent energy parameter of potential function
Source: Sci Rep. 2023 Mar 24;13:4842. doi: 10.1038/s41598-023-31998-7 (PMC10039086; doi:10.1038/s41598-023-31998-7)
Supplement: Supplementary file 4 — Supplementary Information 4. [file 41598_2023_31998_MOESM4_ESM.pdf]

# Supporting Information

# Molecular Theory of Corresponding States

# Critical Temperature Shift Modeling of Confined Fluids Using Pore-Size-Dependent Energy Parameter of Potential Function

by

Mohammad Humand, Mohammad Reza Khorsand Movaghar*

Correspondence: m.khorsand@aut.ac.ir

Department of Petroleum Engineering

Amirkabir University of Technology

Tehran, Iran

Here we aim to explain why $\varepsilon_k$ and critical temperature are in direct relation to each other, and why $\varepsilon_k$ emulates the behavior of $T_c$ when it is decreased because of the confinement. Since John. M. Prausnitz has explained this relation perfectly [1], we bring their discussions on "Molecular Theory of Corresponding States" where a relation is established between the microscopic and microscopic theories of corresponding states using the concept of Canonical Partition Function:

---

## Molecular Theory of Corresponding States

Classical or *macroscopic theory of corresponding states* was derived by van der Waals based on his well-known equation of state. It can be shown, however, that van der Waals' derivation is not tied to a particular equation but can be applied to any equation of state containing two arbitrary constants in addition to gas constant *R*.

From the principle of continuity of the gaseous and liquid phases, van der Waals showed that at the critical point

$$\left(\frac{\partial P}{\partial v}\right)_T = \left(\frac{\partial^2 P}{\partial v^2}\right)_T = 0 \qquad (4\text{-}62)$$

These relations led van der Waals to the general result that for variables υ (volume), *T* (temperature), and *P* (pressure) there exists a universal function such that

$$F\left(\frac{v}{v_c}.\frac{T}{T_c},\frac{P}{P_c}\right) = 0 \qquad (4\text{-}63)$$

is valid for all substances; subscript *c* refers to the critical point. Another way of stating this result is to say that, if the equation of state for any one fluid is written in reduced coordinates (i.e., $\upsilon/\upsilon_C$, $T/T_C$, $P/P_c$), that equation is also valid for any other fluid.

Classical theory of corresponding states is based on mathematical properties of the macroscopic equation of state. *Molecular* or *microscopic theory of corresponding states*, however, is based on mathematical properties of the potential-energy function.

Intermolecular forces of a number of substances are closely approximated by the inverse-power potential function given by Eq. (4-24). The independent variable in this potential function is the distance between molecules. When this variable is made dimensionless, the potential function can be rewritten in a general way such that the dimensionless potential is a *universal function F* of the dimensionless distance of separation between molecules:

$$\frac{\Gamma_{ii}}{\varepsilon_i} = F\left(\frac{r}{\sigma_i}\right) \qquad (4\text{-}64)$$

where $\varepsilon_i$ is an energy parameter and $\sigma_i$ is a distance parameter characteristic of the interaction between two molecules of species i. For example, if function *F* function *F* is given by the Lennard-Jones potential, then $\varepsilon_i$ is the energy (times minus one) at the potential-energy minimum, and $\sigma_I$ is the distance corresponding to zero potential energy. However, Eq. (4-64) is not restricted to the Lennard-Jones potential, nor is it restricted to an inverse-power function as given by Eq. (4-24). Equation (4-64) merely states that the reduced potential energy ($\Gamma_{ii}/\varepsilon_i$) is some universal function of the reduced distance ($r/\sigma_i$).

Once the potential-energy function of a substance is known, it is possible, at least in principle, to compute the macroscopic configurational properties of that substance by the techniques of statistical mechanics. Hence a universal potential-energy function, Eq. (4-64), leads to a universal equation of state and to universal values for all reduced configurational properties.

To obtain macroscopic thermodynamic properties from statistical mechanics, it is useful to calculate the *canonical partition function* of a system depending on temperature, volume, and number of molecules. For fluids containing small molecules, the partition function Q is expressed as a product of two factors,

$$Q = Q_{int}(N,T)Q_{trans}(N,T,V) \tag{4-65}$$

where the translational contributions to the energy of the system are separated from all others, due to other degrees of freedom such as rotation and vibration. It is assumed that contributions from rotation and vibration depend only on temperature. These contributions are called *internal* because (by assumption) they are independent of the presence of other near-by molecules.

In the classical approximation, the translational partition function, $Q_{trans}$, splits into a product of two factors, one arising from the kinetic energy and the other from the potential energy. For a one-component system of *N* molecules, $Q_{trans}$ is given by

$$Q_{trans} = \left(\frac{2\pi mkT}{h^2}\right)^{3N/2} \frac{1}{N!}\int_V \int exp\ \left[-\frac{\Gamma_t(r_1,\ldots,r_N)}{kT}\right] dr_1 \ldots dr_N \tag{4-66}$$

where m is the molecular mass, k is Boltzmann's constant, h is Planck's constant, and $\Gamma_t$ ($r_1, \ldots, r_N$) is the potential energy of the entire system of N molecules whose positions are described by vectors $r_1, \ldots, r_N$. For a given number of molecules and known molecular mass, the first factor depends only on the temperature. The second factor, called the *configurational integral*, $Z_N$, depends on temperature and volume:

$$Z_N = \int_V \int exp \left[-\frac{\Gamma_t(r_1,\ldots,r_N)}{kT}\right] dr_1 \ldots dr_N \quad (4\text{-}67)$$

Hence the configurational part provides the only contribution that depends on intermolecular forces. However, $Z_N$ is *not* unity for an ideal gas ($\Gamma_t$=0). For an ideal gas, $Z_N^{id} = V^N$.

The equation of states is obtained from Q (see App. B):

$$P = kT\left(\frac{\partial \ln Q}{\partial V}\right)_{T,N} = kT\left(\frac{\partial \ln Z_N}{\partial V}\right)_{T,N} \quad (4\text{-}68)$$

The equation of state depends only on $Z_N$ when Eqs. (4-65) and (4-66) are valid. Therefore, the main problem in applying statistical mechanics to real fluids lies in the evaluation of the configurational partition function.

There are four assumptions that lead to the *molecular theorem of corresponding states*, clearly stated by Pitzer (1939) and Guggenheim (1945). They are:

1. The partition function is factored according to Eq. (4-65), where $Q_{int}$ is independent of the volume per molecule.
2. The classical approximation Eq. (4-66) is used for $Q_{trans}$.

3. The potential energy $\Gamma_t$ is represented as the sum of the interactions $\Gamma_{ij}(r_{ij})$ of all possible pairs of molecular. For a given *ij* pair, $\Gamma_{ij}$ depends only on the distance $r_{ij}$ between them:

$$\Gamma_t = \sum_{i<j} \Gamma_{ij}(r_{ij}) \tag{4-69}$$

4. The potential energy of a pair of molecules, reduced by a characteristic energy, is represented as a universal function of the intermolecular distance, reduced by a characteristic length, i.e., Eq. (4-64).

Assumptions 3 and 4 are substituted into the configurational partition function. Further, we use reduced coordinates obtained by dividing the three-dimensional position vectors, $r_1$, …, $r_N$ by the scale factor $\sigma^3$. Thus

$$Z_N = \sigma^{3N} \int_{V/\sigma^3} \int \exp\left[-\frac{\varepsilon}{kT} \sum_{i<j} F\left(\frac{r_{ij}}{\sigma}\right)\right] d\left(\frac{r_1}{\sigma^3}\right) \dots d\left(\frac{r_N}{\sigma^3}\right) \tag{4-70}$$

Apart from the factor $\sigma^{3N}$, the configurational integra. Depends only on *N*, on the reduced temperature $kT/\varepsilon$, and on the reduced volume $V/\sigma^3$ (through the limits of the integral):

$$Z_N = \sigma^{3N} Z_N^*\left(\frac{kT}{\varepsilon}, \frac{V}{\sigma^3}, N\right) \tag{4-71}$$

where $Z_N^*$ is a universal function.

Because configurational Helmholtz energy is given by

$$A^{conf} = -kT \ln Z_N \tag{4-72}$$

and because $A^{conf}$ is an extensive property (proportional to *N*), we have

$$A^{conf} = N\Psi(T, v) \tag{4-73}$$

where the function Ψ depends only on the intensive variables *T* and $\upsilon = V/N$.

Equations (4-72) and (4-73) imply that the configurational integral must be of the form

$$Z_N = \sigma^{3N}\left[z^*\left(\frac{kT}{\varepsilon}, \frac{V}{N\sigma^3}\right)\right]^N \tag{4-74}$$

where function $z^*$ depends only on intensive variables. Substitution of Eq. (4-74) into Eq. (4-68) gives the equation of state

$$\frac{P}{NkT} = \left(\frac{\partial \ln z^*}{\partial V}\right)_{T,N} \tag{4-75}$$

Introducing the reduced variables

$$\tilde{T} = \frac{kT}{\varepsilon} \qquad \tilde{v} = \frac{V}{N\sigma^3} \qquad \tilde{P} = \frac{P\sigma^3}{\varepsilon} \tag{4-76}$$

we find that $z^*$ is a function of $\tilde{T}$ and $\tilde{v}$ [Eq. (4-74)]; the equation of state becomes

$$\tilde{P} = F^*(\tilde{T}, \tilde{v}) \tag{4-77}$$

where $F^*$ is universal function. The nature of this function depends only on the nature of the potential function $\Gamma_{ij}$ in Eq. (4-69).

Equation (4-77) expresses the *molecular* (or *microscopic*) *theory of corresponding states.* This theory is analogous to the macroscopic theory of corresponding states expressed by Eq. (4-63); the difference lies in the reducing parameters.

The reduced quantities [Eq. (4-76)] are defined in terms of macroscopic variables *T, V, P, N* and molecular parameters ε and σ. The use of molecular parameters is important in the extension of the theorem of corresponding states to mixtures.

To relate the *macroscopic* and the *microscopic* theories of corresponding states, it is desirable to establish a connection between the parameters of one theory and those of the other. In the microscopic theory, there are two independent parameters: an energy parameter and a distance parameter. In the macroscopic theory, there appear to be three - $\upsilon_c$, $T_c$, and $P_c$ – but only two of these are independent because, according to the theory, the compressibility factor at the critical point ($z_c=P_c\upsilon_c/RT_c$) is the same for all fluids.

The connection between the macroscopic and the microscopic theories of corresponding states can be established by substituting Eq. (4-77) in to the relations given by Eq. (4-62). It he follows that the macroscopic critical properties $\upsilon_c$, $T_c$ and $P_c$ are related to the molecular parameters ε and σ by

$$\frac{\varepsilon}{k} = c_1 T_c \qquad (4\text{-}78)$$

$$\frac{2}{3}\pi N_A \sigma^3 = c_2 v_c \qquad (4\text{-}79)$$

$$\frac{\varepsilon}{\sigma^3} = c_3 P_c \qquad (4\text{-}80)$$

where $N_A$ is Avogadro's constant ($\upsilon_c$ is per mole) and $c_1$, $c_2$, and $c_3$ are universal constants. For simple nonpolar molecules, i.e., those nonpolar molecules having a small number of atoms per molecule, these relations have been found **empirically** for the case where the generalized function *F* is replaced by the Lennard-Jones (12-6) potential (Hirschfelder *et al.*, 1964). For that particular case, we have, approximately,

$$c_1 = 0.77 \qquad c_2 = 0.75$$
$$c_3 = 7.42 \qquad z_c = \frac{2\pi}{3}\frac{c_1}{c_2 c_3} = 0.29 \tag{4-81}$$

Since the critical temperature is a measure of the kinetic energy of the fluid at a characteristic state (where the liquid and gaseous states become identical), the simple proportionality between energy parameter ε and critical temperature $T_c$ is reasonable. Similarly, the critical volume reflects the size of the molecules; hence, the proportionality between distance parameter $\sigma^3$ and the critical volume is also reasonable. The proportionality of the critical pressure to the ratio $\varepsilon/\sigma^3$ follows because, according to the theory, the compressibility factor $z_c$ is the same for all fluids.

---

You can see that, the critical point is known as the characteristic state, where the liquid and gaseous phases of a substance become identical. At this point, the critical temperature is a measure of kinetic energy, therefore a relation between $\varepsilon_k$ and $T_c$ is quite expectable and reasonable. From the foregoing explanations, knowing that $T_c$ and $\varepsilon_k$ are of one type, and based on the shift of critical temperature of confined fluids, we can infer that the energy parameter of the potential function would accordingly shift. In other words, as a component

has different critical temperature values at different pore radii, there might be also a possibility for $\varepsilon_k$ to vary in pore size which is designated as $\varepsilon_{kp}$ herein.

So why manipulating $\varepsilon_k$ rather than $\sigma_k$ or $a_k$? Along the lines of $\varepsilon_k$ and $T_c$, there is a relation between the size parameters $a_k$ or $\sigma_k$ and the critical molar volume ($v_c$), because the latter represents the molecular size. The point is, however, that $v_c$ of a bulk fluid does not really differ from the confined one. Not only this claim is theoretically conspicuous, but also experiments have made the same assertion as well. Even if there were any shift of $v_c$ regarding pore shrinking, it would be irrelevant to choose a distance parameter for calibrating an energy parameter ($T_c$). Thus, $\varepsilon_k$ (or in fact $\varepsilon$) seems to be the only choice for manipulation.

## References:

[1] J.M. Prausnitz, R.N. Lichtenthaler, E.G. De Azevedo, Molecular thermodynamics of fluid-phase equilibria, Pearson Education1998.
